# Unveiling host-genetic drivers of caecal microbial communities in chickens through genome-wide association studies

**DOI:** 10.3389/frmbi.2025.1539923

**Published:** 2025-02-18

**Authors:** Ankit Hinsu, Xiaoxia Dai, Christos Dadousis, Melanie Hay, Bruno Fosso, Matteo Crotta, Ramesh Pandit, Javier Guitian, Fiona Tomley, Prakash Koringa, Chaitanya Joshi, Damer Blake, Androniki Psifidi

**Affiliations:** ^1^ Clinical Sciences and Services, Royal Veterinary College, Hatfield, United Kingdom; ^2^ School of Health Sciences, University of Surrey, Surrey, United Kingdom; ^3^ Veterinary Health Innovation Engine, School of Veterinary Medicine, University of Surrey, Surrey, United Kingdom; ^4^ Pathobiology and Population Sciences, Royal Veterinary College, Hatfield, United Kingdom; ^5^ Department of Biosciences, Biotechnologies and Environment, University of Bari “Aldo Moro”, Bari, Italy; ^6^ Gujarat Biotechnology Research Centre (GBRC), Department of Science and Technology, Government of Gujarat, Gandhinagar, Gujarat, India; ^7^ Department of Veterinary Biotechnology, College of Veterinary Science and Animal Husbandry, Kamdhenu University, Anand, Gujarat, India

**Keywords:** GWAS, WGS, chicken, enterotypes, microbiota, caecal microbiome, immune system

## Abstract

Enteric microbiota plays a crucial role in the health and productivity of poultry, including influences on nutrient absorption, immune function, and pathogen resistance. In this study, we conducted a genome-wide association study (GWAS) to identify host genetic variants associated with selected bacterial genera found in chickens. We used high-density 600K SNP Affymetrix DNA arrays for genotyping, alongside 16S rRNA gene sequencing to profile caecal microbiota from the same individual chickens. A commercial broiler line (Cobb400, n = 300) and an indigenous (Kadaknath, n = 300) chicken breed from India were investigated, allowing for a comprehensive cross-ecotype analysis. Our analysis identified several host-genetic markers and candidate genes associated with the presence and abundance of specific bacterial genera with known pathogenic or commensal roles, and with specific caecal Enterotypes. Whole-genome sequencing data were then used to further investigate candidate regions around significantly associated variants from the high-density DNA array. Of note, we found markers nearby the genes coding for classical complement activation component C1q, ephrin receptors, and other immunity and inflammatory responses as well as genes coding for products associated with vitamin and co-factor metabolism. The results underscore the impact that host genetics has on the regulation of the gut microbiota and highlights potential pathways through which host genetic variation influences host-bacterial crosstalk and potentially modulates microbial community structure. These findings contribute to the growing understanding of the genetic basis of host-microbiota interactions and offer new avenues for improving poultry health and productivity through selective breeding strategies targeting the microbiome.

## 1 Introduction

Chickens (*Gallus gallus domesticus*) are a cornerstone of global agriculture, serving as one of the most widely consumed sources of animal protein produced with a smaller environmental footprint over other livestock species (OECD et al., 2024). As global demand for poultry products continues to rise, maintaining the health and productivity of chickens has become increasingly vital for food security and economic stability. A key factor in ensuring the well-being of poultry is the gut microbiome, particularly the caecal microbiota, which plays a crucial role in several physiological processes that impact the overall health and productivity of the host (Sergeant et al., 2014). The chicken caeca harbour a diverse and dynamic community of microorganisms composed of trillions of bacteria, archaea, fungi, and viruses, all existing in a delicate equilibrium. The caecal microbiota is integral to digestion and absorption of nutrients, modulation of the immune responses, and therefore protection against pathogens (Pandit et al., 2018).

In chickens, the caecal microbiota is primarily involved in the fermentation of complex carbohydrates that escape digestion in the upper gastrointestinal tract. Through this fermentation process, the microbiota produces short-chain fatty acids (SCFAs) such as acetate, propionate, and butyrate, which are absorbed by the host and serve as significant sources of energy (Gilroy et al., 2021). In addition to SCFAs, the microbiota synthesises essential vitamins, including vitamin K and certain B vitamins, which are crucial for multiple metabolic functions (Sergeant et al., 2014). These microbial activities not only enhance the nutritional value of the diet but also contribute to overall health and growth performance, making the caecal microbiome a critical factor in poultry production (Dittoe et al., 2022).

Beyond its nutritional contributions, the caecal microbiota plays a pivotal role in protecting the host from infectious organisms. The microbial community acts as a barrier by competing with pathogens for nutrients and attachment sites, producing antimicrobial compounds, and modulating the host’s immune responses (Awad et al., 2009). However, the caeca are also known reservoirs for zoonotic pathogens such as *Campylobacter*, pathogenic *Escherichia coli*, *Salmonella*, and *Helicobacter* (Kaakoush et al., 2014). These pathogens can persist in the caecal environment and be transmitted to humans through the consumption or handling of contaminated poultry products, posing significant public health risks (Berry and Wells, 2016). The caecal microbiota is also a potential reservoir of antimicrobial resistance genes (ARGs) that can be transferred to pathogenic bacteria, exacerbating the challenge of controlling infections in animals and humans (Hedman et al., 2020).

The composition of the caecal microbiota is highly dynamic and is influenced by a multitude of factors, including geographic location, environmental conditions, diet and feed additives, age, and farming practices (Kers et al., 2018). Among these, diet is a particularly important determinant of microbial composition. The composition and quality of feed, including the presence of prebiotics, probiotics, other dietary supplements, and toxins, can significantly alter the microbial ecosystem within the caeca (Li et al., 2020). In addition to environmental and dietary factors, host genetics also play a critical role in shaping enteric microbial populations. Recent studies have highlighted the significant impact of host genetic makeup on the composition and function of gut microbiota (Benson et al., 2010). Genetic variations in the host can influence several aspects of microbiome structure, including the abundance of specific microbial taxa, the overall diversity of the microbial community, and the functional capabilities of the microbiota (Kurilshikov et al., 2017). These findings suggest that the microbiome is, to some extent, heritable and that selective breeding could be a potential strategy for optimising gut health in poultry and thereby overall health, welfare and productivity. Moreover, the relationship between host genetics and the gut microbiota is bidirectional. Not only do host genetics influence the microbiome, but the microbiome also affects the expression of host genes, particularly those involved in immune responses and metabolic processes (Kers et al., 2018). This intricate host-microbiome crosstalk highlights the complexity of the gut ecosystem and underscores the importance of considering both genetic and environmental factors in the management of poultry health. Understanding the genetic basis of these interactions will be valuable when developing targeted interventions that can enhance the health, productivity, and disease resistance of chickens.

In this study, we aimed to identify host genetic variation associated with selected caecal bacterial genera that are known to play either pathogenic, beneficial or commensal roles in the gut microbiome of chickens. Leveraging a comprehensive approach, we used host genotype data obtained through a genome-wide high-density DNA array, alongside microbiota data derived from 16S rRNA gene sequencing, both collected from the same individual chickens (commercial broilers, Cobb400 and indigenous chickens, Kadaknath from India). Our previous study that compared microbiome composition in two indigenous Indian breeds (Kadaknath and Aseel) and two commercial broiler lines (Cobb400 and Ross 308) demonstrated comparatively greater effects of location than breed/variety in shaping the chicken caecal microbiome (Pandit et al., 2018). Nevertheless, in closer examination of variation in microbiome structure between the commercial broilers and indigenous breeds, several key biomarkers associated with chicken varieties were identified, indicating further scope for quantitative genetic analysis. Moreover, the microbiome dataset used in this work was previously described in our other work, which explored the concept of caecal Enterotypes - a distinct state of microbiota structure within the caeca (Hay et al., 2023). Originally proposed in 2011, the concept of enterotypes arose from the finding that human gut microbiomes could be assigned to one of a small number of stable states (enterotypes) based on the relative proportions of dominant bacterial taxa (Arumugam et al., 2011). The concept was later applied to other animal and bird species including chickens (Moeller et al., 2012; Wang et al., 2014; Mach et al., 2015; Ramayo-Caldas et al., 2016; Yuan et al., 2020). Enterotypes are shaped over time by dietary intake and demonstrate resilience to temporary fluctuations in diet (Arumugam et al., 2011). In line with other studies, our previous study observed the presence of 3 distinct Enterotypes differing by the ratio of *Firmicutes* to *Bacteroides* with a decreasing ratio from Enterotype 1 to 3 (Hay et al., 2023). In the current study, we extend this concept by using Enterotype as one of the phenotypes for genome wide association studies (GWAS), thereby linking specific host genetic variants to the shaping of specific microbial community structures. Additionally, we analysed host whole-genome sequencing (WGS) data at high coverage (40 X) to delve deeper into candidate genomic loci that show strong associations with the caecal microbiota or Enterotypes in the GWAS. By focusing on the genetic determinants of caecal microbial composition, this study aims to provide new insights into the complex interactions between the host and the gut microbiome that will advance understanding of how host genetic variation influences the microbiome and exploration of the potential for selective breeding strategies that leverage these insights to improve the health and productivity of poultry.

## 2 Materials and methods

### 2.1 Ethical approval

The work was conducted with permission from the Ethical Review Panel of Anand Agricultural University (AAU) (now Kamdhenu University) and the Clinical Research Ethical Review Board (CRERB) of the Royal Veterinary College under the reference URN 2014 1280. Participating farmers were informed of the objectives of the study and written consent was obtained.

### 2.2 Collection of samples

Two chicken lines, Cobb400 and Kadaknath, were selected for the study due to their distinct genetic backgrounds and production traits. Cobb400, a widely used commercial broiler in the study region, has been intensively selected for rapid growth and feed efficiency. In contrast, Kadaknath, an indigenous Indian slow growing black-boned breed, is known for disease resilience (Ramasamy et al., 2010). Samples from 600 chickens were collected from the farms/backyard production systems in Gujarat, a western state of India. The sampled chickens included 300 commercial Cobb400 broiler and 300 indigenous Kadaknath chickens. The birds were sampled from farms rearing both lines together (n = 30, 5 birds sampled of each line per farm), broiler-only (n = 15, 10 birds sampled per farm) or Kadaknath-only farms (n = 15, 10 birds sampled per farm), The birds were sacrificed between 35 and 42 days of age and blood was collected onto NucleoSave cards (Macherey-Nagel, Germany) for genomic DNA extraction. Caecal pouches were surgically removed post-mortem to collect the caecal content in Qiagen RNAprotect Bacteria reagent (1:1 ratio) for microbiome analysis. At each farm/site, detailed metadata related to location and farming/management practices was also collected to control for environmental variation in the genetic studies. The detailed description of the experimental design, including the location of sites and the metadata for each site is outlined in detail in our previous studies (Hinsu et al., 2018; Hay et al., 2023).

### 2.3 Sample processing and data generation

#### 2.3.1 DNA extraction and sequencing

Total DNA was extracted from caecal content using a Qiagen DNeasy Stool Mini Kit (Qiagen, Germany) following the manufacturer’s instructions with minor modifications (Pandit et al., 2018). Briefly, 200 µl of sample was mixed with 1 ml InhibitEX buffer and incubated at 80°C for 10 minutes. The mixture was centrifuged and 600 µl of supernatant was processed as per the protocol. Around 12.5 ng DNA was used to amplify V3-V4 regions of the 16S rRNA gene using 341F and 785R primers and prepare libraries as mentioned in the Illumina 16S library preparation guide (Illumina Inc., USA). The libraries were sequenced on the Illumina MiSeq platform using 300x2 paired-end chemistry. A total of nine sequencing runs were set to accommodate all the samples.

#### 2.3.2 Host genotyping

Chicken’s genomic DNA was extracted from NucleoSave cards using the GenSolve DNA kit (GenTegra, USA) as per the manufacturer’s instructions. Genomic DNA from all 600 samples was submitted to Edinburgh Genomics to genotype using the Axiom Genome-wide Chicken Array (ThermoFisher Scientific, USA). The Axiom Genome-wide Chicken Array contains around 620,000 SNP markers (Kranis et al., 2013).

#### 2.3.3 Whole-genome sequencing (WGS)

A subset of 31 chicken DNA samples (Cobb400 = 13, Kadaknath = 18) were selected based on specific genera abundance such as *Campylobacter* and *Escherichia.Shigella* to represent chickens in the extremes of the phenotypes for whole genome sequencing. Libraries were prepared using Illumina TruSeq DNA Nano kits (Illumina Inc., USA) and sequenced on the Illumina HiSeq platform using 2x150 bp chemistry to generate average 40x genome coverage per sample. The library preparation and sequencing were outsourced to Edinburgh Genomics (UK).

### 2.4 Data analysis

#### 2.4.1 16S rRNA sequencing data analysis

The 16S rRNA gene sequencing data was analysed using the DADA2 package (Callahan et al., 2016). Briefly, the data was quality filtered, denoised, paired reads were merged, and chimera-checked to generate the final Amplicon Sequence Variants (ASV) table using the default parameters as given in the DADA2 tutorial (https://benjjneb.github.io/dada2/tutorial.html). In line with DADA2 recommendations, samples from each flow cell were processed individually and then merged at a later stage before generating the ASV table. ASVs were assigned taxonomy using SILVA v132 and phylogeny was generated within QIIME2 (Quast et al., 2013; Bokulich et al., 2018; Bolyen et al., 2019). An additional step of clustering sequences with 99% similarity was performed to reduce the number of ASVs for taxonomic classification within QIIME2. The ASV table, phylogenetic tree, taxonomy information and metadata were merged to generate a phyloseq object which was then used for other downstream analysis (McMurdie and Holmes, 2013). The samples were rarefied to 10,000 reads and samples with less than 10,000 reads were removed from the analysis. The ASV table was agglomerated at genus level to obtain genera level abundance.

From the detected genera, *Bacillus*, *Campylobacter*, *Cloacibacillus, Eisenbergiella, Enterococcus*, *Escherichia*.*Shigella*, *Helicobacter*, *Lactobacillus*, *Parasutterella* and *Sutterella* were selected as phenotypes for GWAS. The choice of genera was made based on their involvement and role in chicken caecal microbiota (Oakley et al., 2014; Hiippala et al., 2016; Lim et al., 2017; Clavijo and Florez, 2018; Zhang et al., 2021; Derqaoui et al., 2022; Chen et al., 2023; Ge et al., 2023). For instance, *Campylobacter* and *Helicobacter* are common zoonotic pathogens from chickens, while *Bacillus* and *Lactobacillus* are key commensals/beneficial microbes for chicken.

Genera detected in 30%-60% of samples (*Bacillus*, *Campylobacter, Cloacibacillus, Enterococcus, Escherichia*-*Shigella* and *Sutterella*) were analysed as binary traits (present/absent), while those detected in >60% of samples (*Eisenbergiella*, *Helicobacter*, *Lactobacillus* and *Parasutterella*) were analysed as continuous traits in subsequent genetic analysis. The relative abundance of genera analysed as continuous traits was normalised by rank-based inverse normal transformation using the GenABEL package v1.8-0 in R (Aulchenko et al., 2007).

Additionally, the classified Enterotypes from our previous study were also used as phenotypes for GWAS (Hay et al., 2023). Enterotypes refer to the distinct clusters of enteric microbiota based on the relative abundance of different bacterial species. Enterotypes were classified by clustering the AIT distance matrix of genus-level abundance using partition around medoids (PAM) clustering algorithm. Please refer (Hay et al., 2023) for the detailed methodology of Enterotype classification.

#### 2.4.2 Genotypic data

PLINK v1.9 (https://www.cog-genomics.org/plink/) was used for quality control of the genotypic data: minor allele frequency greater than 5%, sample call rate greater than 90% and a Hardy-Weinberg equilibrium p-value less than 10^-6^ (flags: –maf 0.005 –geno 0.1 –hwe 0.000001) (Purcell et al., 2007). The filtered variants were used to generate genomic relationship matrix which was then used to perform Principal Component Analysis and plotted using R. SNP positions of the markers were remapped to the galGal6 (GRCg6a) chicken genome assembly using the LiftOver remapping tool (https://genome.ucsc.edu/cgi-bin/hgLiftOver). Updated SNP positions on galGal6 were used for all downstream analysis.

#### 2.4.3 Metadata information

Additional information for each farm/site was collected in the form of detailed questionnaire. The questionnaire included detailed information covering the farm description, farm location, surrounding geographical features, farming practices and other factors potentially affecting microbiota like other animals/chickens on farms. More details are available from our previous publication (Hay et al., 2023). Multiple correspondence analyses (MCA) of bird and environmental farm variables were performed to identify key components explaining the phenotypic variance in recorded metadata using FactoMineR package in R (Lê et al., 2008). The first four MCA axes, which explained more than 70% of variation, were taken as a covariates for SNP-based heritability estimates and genome-wide association study (GWAS) analyses.

#### 2.4.4 Heritability estimate & GWAS

QC filtered variants were used to estimate SNP-based heritability for each selected genera and Enterotypes individually using the genomic relatedness based restricted maximum-likelihood method (GREML) implemented within GCTA v.1.94.beta (Yang et al., 2011). The genomic relationship matrix among individuals (GRM) was produced using ‘–make-grm’ flag as


$${ɡ}_{ij}=\frac{1}{N}\sum_{v=1}^{N}\frac{\left(x_{iv}-2\bar{p_{v}}\right)(x_{jv}-2\bar{p_{v})}}{2\bar{p_{v}(1-\bar{p_{v}}})}$$

in which g_ij_ represents the estimated genetic relationship between chicken i and j; x_iv_ and x_jv_ are the counts of the reference alleles in chicken i and chicken j, representatively; $\bar{p_{v}}$ is the frequency of the reference allele in the population; and N is the total number of SNPs. SNP-based heritability, i.e. the proportion of total phenotypic variance attributed to genetic variation, was quantified using restricted maximum likelihood analysis (‘–reml’) function in a model that included the first four MCA axes and breed as covariates to account for potential environmental effects.

GWAS analyses were performed using the Genome-Wide Efficient Mixed Model Association (GEMMA) algorithm v0.98 (Zhou and Stephens, 2012) for each selected genera and Enterotype as a separate trait. GEMMA employs a linear mixed model (LMM) framework that accounts for population structure and relatedness by incorporating a genomic relationship matrix as a polygenic random effect. This approach helps control for confounding due to cryptic relatedness and population stratification, thereby reducing false-positive associations. For each GWAS, chicken breed and the first four MCA components of environmental farm categorical variables were fitted as covariates to account for potential environmental influences on microbial composition. The Wald test, implemented within GEMMA, was used to assess the significance of SNP associations. To control for multiple testing, a Bonferroni correction was applied, setting the genome-wide significance threshold at 1.19 × 10^-7^ (0.05/418,665) and a suggestive genome-wide, counting for one false discovery per genome scan, threshold of 2.39 × 10^-6^ (1/418,665). The GWAS results were visualised using Manhattan plots and quantile-quantile (Q-Q) plots produced using the rMVP R package (Yin et al., 2021).

The 50 kb upstream and downstream regions of the significantly associated SNPs from GWAS results were considered as the candidate region for further analysis. The threshold for genomic regions was determined based on the values derived from the LD decay in both breeds (see Results section). All the genes located in the candidate regions were identified using the BiomaRt R package using Galgal6a database from Ensembl v106 annotations (Durinck et al., 2009). The extracted gene list was used as the input to perform enrichment analysis of KEGG pathways using Clusterprofiler R package (Yu et al., 2012). The results were corrected using Benjamini-Hochberg correction with significance threshold set at P< 0.1 to account for the smaller number of genes.

#### 2.4.5 Whole genome sequencing data analysis

The quality of the sequencing reads was assessed with FastQC v0.11.9 software (https://www.bioinformatics.babraham.ac.uk/projects/fastqc) and QC was performed with Trimmomatic v0.39 (Bolger et al., 2014). QC-pass data was mapped to the galGal6 (GRCg6a) chicken genome assembly using the Burrows-Wheeler Alignment tool (BWA v0.7.10) (Li and Durbin, 2009) and variant calling was performed following the best practice for the Genome Analysis Toolkit with GATK v4.1.6 (McKenna et al., 2010). The vcf file was further filtered according to hard-filter thresholds suggested within the GATK pipeline, and only bi-allelic SNPs were used for downstream analysis. Linkage disequilibrium (LD) decay from the WGS data was estimated by calculating r^2^ and plotted for each population using PopLDdecay with default parameters (Zhang et al., 2019).

All the variants within the candidate regions based on the GWAS results were extracted from the WGS data using bcftools v1.9 (Danecek et al., 2021). The extracted variants were annotated using VEPv110 with chicken GRC6a assembly from Ensembl genes 106 (McLaren et al., 2016). Further, the variants with a predicted ‘HIGH or ‘MODERATE’ impact were filtered for further interrogation (Vaser et al., 2016). These HIGH or MODERATE impact variants included all variants with deleterious effects predicted by SIFT. The variants were further removed if variant only had a single genotype, or less than 3 samples were observed for any genotype. The filtered variants were associated with respective phenotype/trait using linear model in R (lm() function) and p-values adjusted using Benjamini-Hochberg (BH). The phenotype/trait was taken as dependent variable, and individual genetic variants were tested as fixed effects while adjusting for the effects of covariates (the first four MCA components of environmental farm categorical variables and breed). The BH-adjusted p-value threshold of 0.05 was considered as threshold for significance of associations. The LD between all pairs of these tested variants were calculated using GWLD package in R (Zhang et al., 2023).

## 3 Results

The study included 600 chickens: 300 Cobb400 broilers, bred for meat production and 300 Kadaknath, a black-boned indigenous Indian breed, farmed for meat and eggs. All chickens were sampled from Gujarat, the Western-most state of India. While the two varieties have different growth rates, birds were sampled at the same age range of 35-42 days to avoid age bias. Data from both chicken breeds were analysed together to identify genetic variation associated with microbiome structure and genera abundance across chicken breeds.

### 3.1 Microbiome data

Each caecal microbiome was studied by sequencing the V3-V4 region of the 16S rRNA gene across nine separate Illumina sequencing runs. The data was analysed using the DADA2 pipeline, resulting in 9217 Amplicon Sequence Variants (ASVs) from 600 samples. After rarefying at 10,000 reads and removing samples failing QC in host genotype data, 6326 ASVs remained from 559 chicken samples which included 292 Cobb400 and 267 Kadaknath chickens. Agglomerated abundance per genus was considered as phenotypes for performing GWAS, which showed *Bacteroides* and *Faecalibacterium* as the dominant genera (**Supplementary Figure S1**). Ten genera were selected for their beneficial/commensal or pathogenic/zoonotic roles and analysed as binary or continuous traits in GWAS, depending on their prevalence across the samples (**Table 1**). Additionally, microbiome community state, or “Enterotype” was also used as a phenotype. In our previous study using the same set of samples, three distinct Enterotypes were defined in the caecal microbial community (Hay et al., 2023). In line with other studies, these Enterotypes differed by the ratio of *Firmicutes* to *Bacteroides* with a decreasing ratio from Enterotype 1 to 3. As Enterotypes are stable-states of community, they can provide a better measure for studying host-microbiome crosstalk compared to individual taxa abundance/prevalence. Details regarding the microbiome structure and diversity, differences between Cobb400 and Kadaknath, as well as the classification of Enterotypes, have been thoroughly covered in our previous publications (Pandit et al., 2018; Hay et al., 2023).

**Table 1:** Genome-wide heritability estimated for colonisation by selected bacterial genera and caecal enterotypes using GCTA-GREML.

Trait/Genera	Trait type	h2 (pve)	P-Value
*Bacillus*	Binary	0.000001	0.5
*Campylobacter*	Binary	0.200407	0.000166
*Cloacibacillus*	Binary	0.084353	0.12364
*Eisenbergiella*	Continuous	0.211536	0.00301
*Enterococcus*	Binary	0.332069	0.002271
*Escherichia.Shigella*	Binary	0.028209	0.2102
*Helicobacter*	Continuous	0.273572	8.68E-05
*Lactobacillus*	Continuous	0.103375	0.10777
*Parasutterella*	Continuous	0.138161	0.001808
*Sutterella*	Binary	0.168451	0.000158
*Enterotype*	Mutinomial	0.13	0.3

### 3.2 Genotypic data quality control and multidimensional scaling analysis

All 600 chicken samples were genotyped with the chicken 600K single-nucleotide polymorphism (SNP) Affymetrix array (Kranis et al., 2013). After quality control with PLINK v1.9, 418,665 high-quality SNPs from 559 samples remained for further analysis. As expected, multidimensional scaling analysis (MSA), based on the SNP data, revealed population structure and showed a clear separation between the two chicken breeds (**Supplementary Figure S2**) (Psifidi et al., 2018).

### 3.3 Heritability estimates

Genetic heritability (*h* ^2^) was estimated separately for each selected genus and Enterotype. Heritability for the selected genera ranged from 0 to 0.33, with 6 of the 10 genera exhibiting non-zero estimates (likelihood ratio test, *P* < 0.05) (**Table 1**). *Enterococcus* (*h* ^2^ = 0.33) and *Helicobacter* (*h* ^2^ = 0.27) were the genera with the highest heritability estimates from binary and continuous phenotypes, respectively. Similarly to previous studies, species of the zoonotic genus *Campylobacter* showed significant genetic heritability (*h* ^2^ = 0.20, *P* < 0.01) (Psifidi et al., 2016b; Banos et al., 2020; Psifidi et al., 2021), while very low genetic heritability was observed for *Escherichia.Shigella* (*h* ^2^ = 0.03, *P* = 0.21). Interestingly, for the most prevalent genus *Lactobacillus* (present in 92% of the samples) the heritability was close to zero. For the Enterotypes, a low genetic heritability was estimated (*h* ^2^ = 0.13, *P* < 0.3).

### 3.4 GWAS

**Figure 1:**
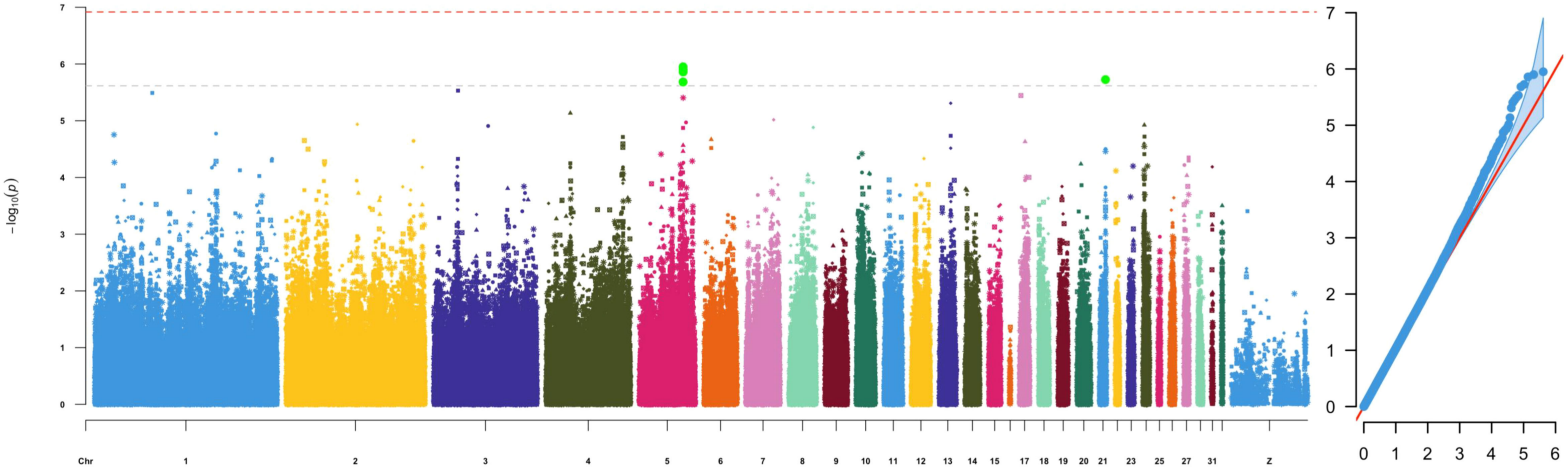
Manhattan plot (left) and QQplot (right) depicting -log_10_(p-value) from the GWAS results with caecal Enterotypes as trait.

Genotypes and phenotypes were compared from 559 birds using a linear mixed model with GEMMA v0.9.8 (Zhou and Stephens, 2012). The GWAS studies identified 7 significant (p < 1.19 × 10^-7^) and 28 suggestive significant (p < 2.39 × 10^-6^) genome-wide level associations between SNPs and the presence of specific gut microbial genera (**Table 2**; **Supplementary Figures S3**–**S12**). Genome-wide significant associations were identified for *Campylobacter* (GGC4: ~39.2Mb, GGC8:~26.9Mb, GGC10:~2.5Mb, GGC25:~2.6Mb)*, Sutterella* (GGC1:~180.3Mb) and *Parasutterella* (GGC1:~90.6Mb) abundance on *Gallus gallus* Chromosomes (GGC) 1, 4, 8, 10 and 25 (**Table 2**). The most significant association (p = 6.28x10^-13^) was observed between *Campylobacter* abundance and a SNP located at 26,982,059 bp on GGC8, which was close to *HOOK1* (Hook Microtubule Tethering Protein 1) gene. Overall, the greatest number of associations were observed for *Campylobacter* and *Cloacibacillus* (9 SNPs, each), followed by *Parasutterella* (5 SNPs) and *Enterococcus* (4 SNPs). There were no significant associations with genera *Bacillus* and *Lactobacillus*, the two most common commensals, which is in accordance with the zero heritability estimated for them. Moreover, we also identified five suggestive genome-wide associations between SNPs and caecal Enterotypes, four of which were closely located on GGC5 (**Table 2**; **Figure 1**). Two sets of associations were observed on GGC5 separated by approximately 74 kb distance. Interestingly, the candidate regions for Enterotypes included genes coding three chains of complement component 1q (i.e.*C1qA*, *C1qB* and *C1qC*).

**Table 2:** Significant SNPs identified by GWAS and candidate genes associated with colonisation by the selected bacterial genera and caecal enterotypes.

Trait/Genera	Chr	Position^a^	EAF^b^	Minor/major allele	Effect size (SE)^c^	*P*-value	Variant present in genes^d^	Closest genes^e^	Association type^f^
*Campylobacter*	2	8,757,131	0.133	A/G	-0.241 (0.046)	1.90E-07	*DNAJB6*		suggestive
*Campylobacter*	3	89,087,094	0.052	G/A	0.342 (0.069)	1.02E-06			suggestive
*Campylobacter*	4	39,239,123	0.094	C/A	0.313 (0.053)	4.91E-09		*UFSP2*	significant
*Campylobacter*	4	72,869,912	0.345	T/C	0.163 (0.03)	5.50E-08			significant
*Campylobacter*	8	26,982,059	0.133	C/T	0.312 (0.042)	6.28E-13		*HOOK1*	significant
*Campylobacter*	10	2,544,683	0.212	C/T	0.176 (0.032)	6.24E-08		*NEO1*	significant
*Campylobacter*	11	7,485,068	0.098	G/A	0.244 (0.05)	1.60E-06		*PHKB*	suggestive
*Campylobacter*	14	8,668,181	0.118	T/C	0.214 (0.043)	9.92E-07	*XYLT1*		suggestive
*Campylobacter*	25	2,654,283	0.108	G/T	0.296 (0.051)	9.01E-09		*BCAN*	significant
*Cloacibacillus*	1	54,516,046	0.358	T/C	-0.2 (0.039)	5.06E-07	*CHST11*		suggestive
*Cloacibacillus*	1	63,705,229	0.494	A/T	-0.15 (0.029)	4.72E-07			suggestive
*Cloacibacillus*	1	103,639,532	0.444	T/C	-0.161 (0.032)	8.72E-07		*MRPL39*	suggestive
*Cloacibacillus*	1	131,150,262	0.383	G/A	-0.167 (0.031)	1.37E-07		*SHOX*	suggestive
*Cloacibacillus*	1	154,591,459	0.468	T/A	-0.151 (0.029)	4.19E-07		*RBM26*	suggestive
*Cloacibacillus*	3	25,624,886	0.085	C/T	-0.301 (0.06)	6.85E-07	*CAMKMT*		suggestive
*Cloacibacillus*	4	52,281,030	0.079	T/A	-0.28 (0.059)	2.23E-06			suggestive
*Cloacibacillus*	24	1,638,713	0.233	T/C	-0.151 (0.031)	1.85E-06		*ZBTB44*	suggestive
*Cloacibacillus*	27	3,611,030	0.138	G/A	-0.228 (0.047)	1.51E-06	*MEIOC*		suggestive
*Eisenbergiella*	14	9,822,602	0.118	A/T	-0.463 (0.097)	2.10E-06		*GRIN21*	suggestive
*Eisenbergiella*	15	8,521,076	0.398	T/C	0.298 (0.059)	7.43E-07		*TBX6*	suggestive
*Enterococcus*	2	72,334,120	0.434	A/C	-0.151 (0.031)	1.35E-06			suggestive
*Enterococcus*	2	73,947,651	0.238	T/A	0.185 (0.038)	1.34E-06			suggestive
*Enterococcus*	12	19,220,163	0.408	T/C	-0.155 (0.032)	1.99E-06		*GRM7*	suggestive
*Enterococcus*	18	8,716,929	0.216	A/T	-0.177 (0.037)	2.23E-06		*SOX9*	suggestive
*Escherichia.Shigella*	8	12,964,841	0.123	A/T	-0.250 (0.05)	7.67E-07		*DPYD*	suggestive
*Escherichia.Shigella*	23	4,347,551	0.119	C/A	0.256 (0.053)	1.95E-06	*AGO1*		suggestive
*Helicobacter*	1	72,755,120	0.064	C/T	0.536 (0.103)	2.84E-07	*PTHLH*		suggestive
*Helicobacter*	7	11,184,189	0.094	T/C	0.397 (0.082)	1.55E-06	*ENSGALG00000045898*		suggestive
*Helicobacter*	23	2,780,356	0.354	C/G	0.273 (0.053)	3.54E-07	*EPB41*		suggestive
*Parasutterella*	1	90,647,255	0.465	A/G	-0.285 (0.049)	9.01E-09		*EPHA3*	significant
*Parasutterella*	1	90,659,508	0.394	A/G	0.269 (0.051)	1.97E-07		*EPHA3*	suggestive
*Parasutterella*	1	188,921,900	0.115	T/C	0.392 (0.074)	1.47E-07	*NAALAD2*		suggestive
*Parasutterella*	3	103,077,236	0.452	C/G	-0.257 (0.05)	3.41E-07			suggestive
*Parasutterella*	4	80,610,041	0.379	G/A	-0.254 (0.053)	2.06E-06		*AFAP1*	suggestive
*Sutterella*	1	180,309,157	0.264	G/C	-0.209 (0.038)	5.83E-08		*GJB6*	significant
Enterotype	5	46,699,728	0.209	T/C	-0.298 (0.061)	1.12E-06		*PAPOLB*	suggestive
Enterotype	5	46,718,518	0.207	G/A	-0.291 (0.061)	2.07E-06		*PAPOLB*	suggestive
Enterotype	5	46,792,605	0.208	T/G	-0.295 (0.061)	1.38E-06	VRK1		suggestive
Enterotype	5	46,795,872	0.212	C/T	-0.296 (0.06)	1.26E-06	VRK1		suggestive
Enterotype	21	6,091,049	0.141	A/G	-0.348 (0.072)	1.88E-06		*C1QA*	suggestive

a Position were derived from remapping to Galgal6 by using LiftOver remapping tool.

b The estimated allele frequency calculated using the data from both breeds.

c Linear regression coefficient beta and standard error (SE) of the minor allele.

d The gene covering the variant from the Ensembl by using the new remapped position from Galgal6. If no gene was covering the variant, the closest gene is mentioned in the next column.

e The physically closet genes were derived from the Ensembl by using the new remapped position from Galgal6. Empty values represent no nearby genes in 250 kb upstream/downstream region.

f Suggestive = GWAS suggestive SNP based on the P-value threshold of 2.39 × 10^-6^. Significant = GWAS significant SNP based on the P-value threshold of 1.19 × 10^-7^.

50 kb upstream/downstream windows around the significant SNPs were considered as candidate regions to search for genes and genetic variants of interest for each of the phenotypes under investigation. The selection of 50 kb windows was based on Linkage Disequilibrium (LD) analysis as described below. Combined, all the candidate regions contained a limited number of protein-coding genes (n = 82) and non-coding RNAs (n = 33) (**Supplementary Table S1**). Consistent with the number of identified associations, most protein-coding genes were observed within the candidate regions for *Campylobacter* abundance (n = 25) followed by *Cloacibacillus* abundance (n = 15) and Enterotype (n = 9).

**Figure 2:**
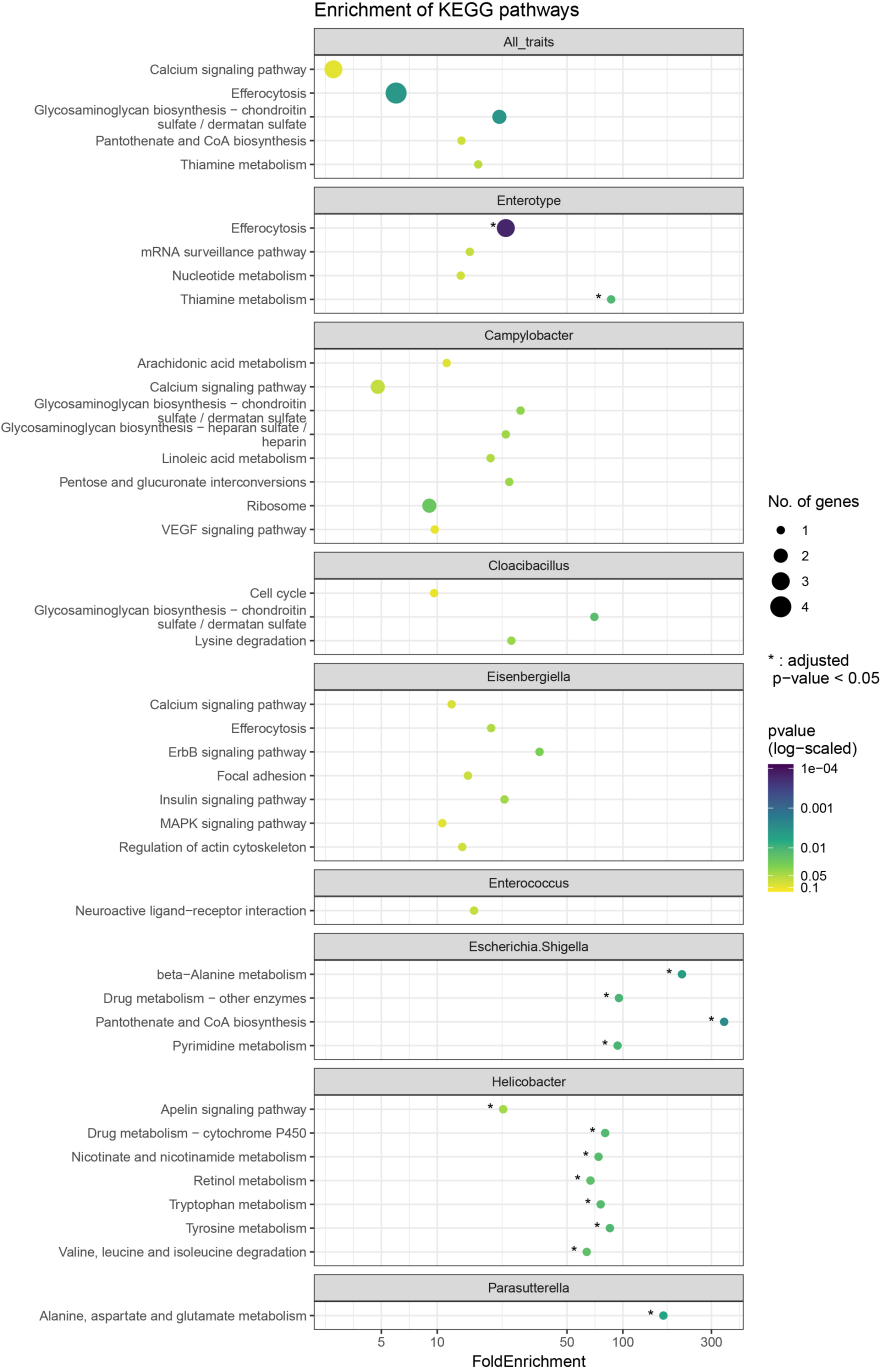
Dot plot showing enriched KEGG pathways from over enrichment analysis. The size of dots corresponds to the number of genes observed for the respective category and the dots are coloured by -log10 of the nominal p-value. * represents the categories which are also significant as per adjusted p-value < 0.05. The top facet shows results from the over enrichment with all the genes taken together.

Enrichment analysis of the gene lists in the candidate regions revealed enrichment for 14 KEGG pathways (Benjamini-Hochberg (BH) adjusted p < 0.05) (**Figure 2**). The most significantly enriched KEGG pathway was efferocytosis (BH adjusted p = 0.0009), related to the Enterotype phenotype. Other pathways with enrichment were part of signal transduction, amino acid metabolism and metabolism of cofactors and vitamins. Specifically, thiamine metabolism (BH adjusted p = 0.035) for Enterotype; apelin signalling pathway (BH adjusted p = 0.044), tyrosine metabolism (BH adjusted p = 0.018), tryptophan metabolism (BH adjusted p = 0.018), valine, leucine and isoleucine degradation (BH adjusted p = 0.018), nicotinate and nicotinamide metabolism (BH adjusted p = 0.018), and retinol metabolism (BH adjusted p = 0.018) for *Helicobacter* abundance; beta-alanine metabolism (BH adjusted p = 0.0096) and pantothenate and CoA biosynthesis (BH adjusted p = 0.0096) for *Escherichia.Shigella* abundance; ErbB signalling pathway (BH adjusted p = 0.105) for *Eisenbergiella* abundance; lysine degradation (BH adjusted p = 0.079) for *Cloacibacillus* abundance; and alanine, aspartate and glutamate metabolism (BH adjusted p = 0.006) for *Parasutterella* abundance.

### 3.5 Whole genome sequencing

**Figure 3:**
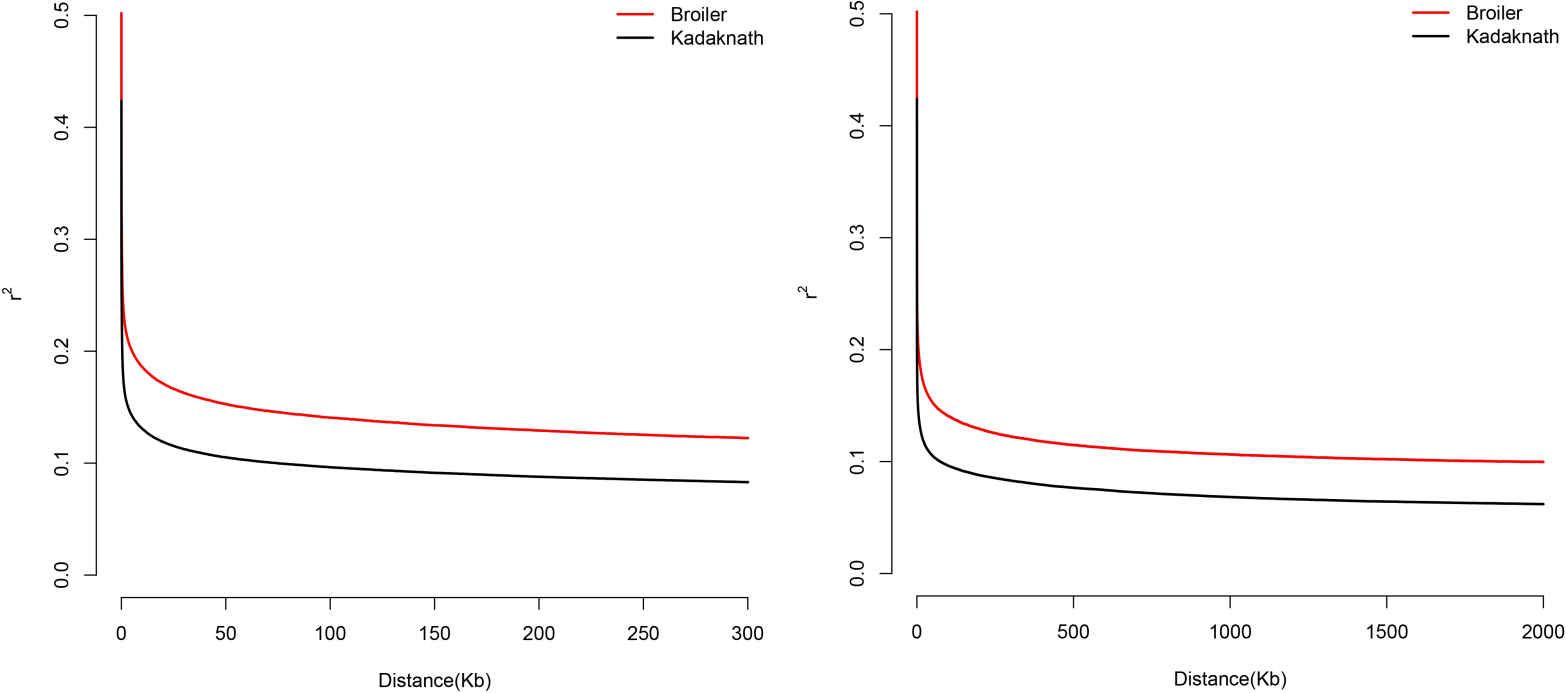
Linkage disequilibrium (LD) decay in Cobb400 (broiler) and Kadaknath chickens calculated from the variants predicted from WGS data. Left plot shows decay in the distance less than 300 kb, right plot shows the same data in the distance 2 Mb.

High coverage (40x) WGS data from 31 selected chickens (Cobb400 = 13, Kadaknath = 18) was processed through the Genome Analysis Toolkit (GATK) v4.1.6 variant-calling pipeline (McKenna et al., 2010), identifying around 20 million variants. Of these, around 17 million biallelic variants were used to calculate LD decay. The LD plot showed r^2^ value below 0.2 at ~ 50 kb distance, wherein it started to flatten (**Figure 3**). The LD decay curve showed higher levels for Cobb400 compared to Kadaknath, which was consistent with the higher selection pressure on commercial broilers. Based on these results, 50 kb was considered the most appropriate window for candidate genomic regions.

**Figure 4:**
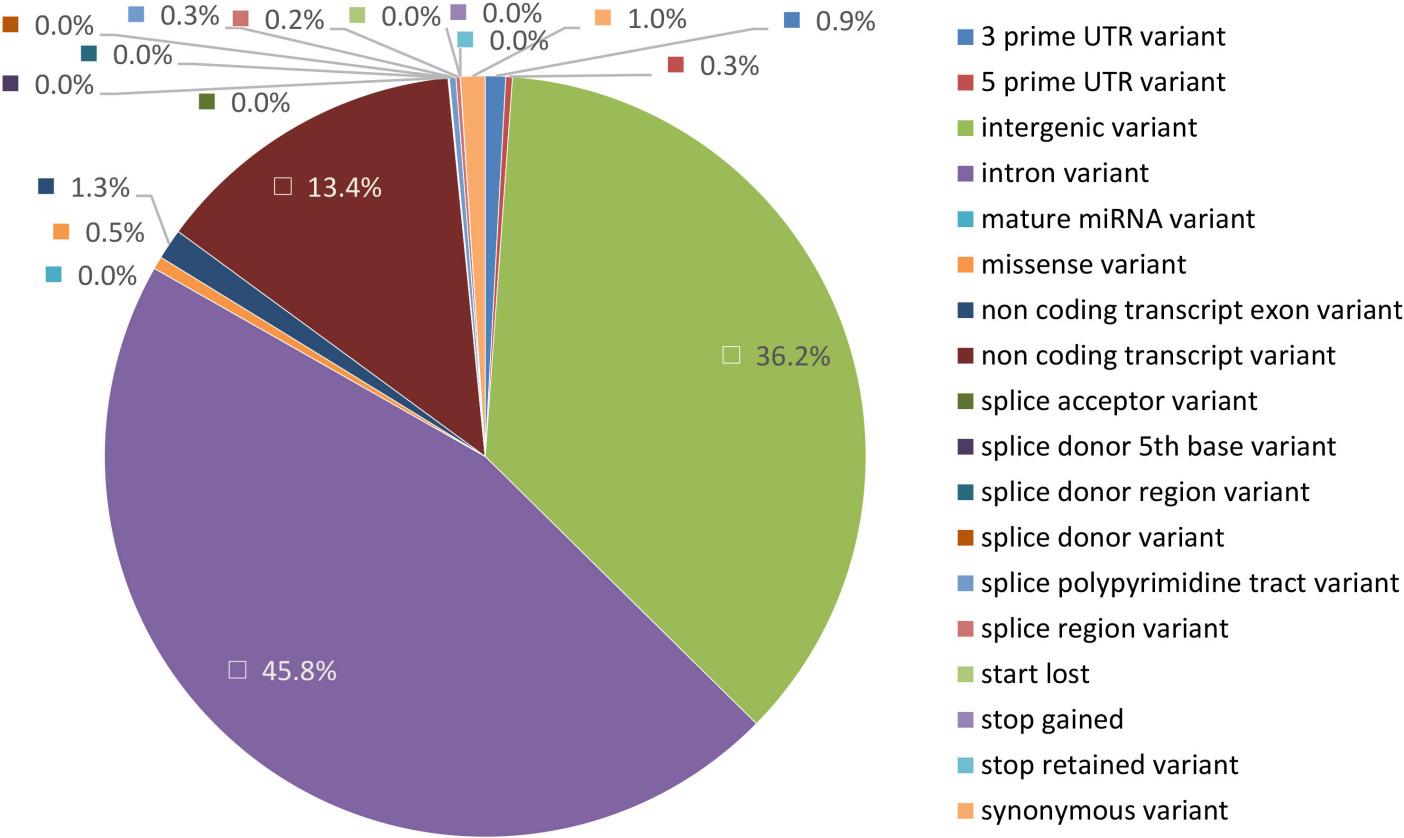
Pie chart representing genetic location of variants from WGS data present in GWAS candidate regions annotated with Variant Effect Predictor (VEP). The proportion represented is from 50,262 variants detected in candidate regions.

To identify genetic variants of interest in these candidate regions we used the variants called from the WGS data. The variants were extracted and annotated with VEP v110 (McLaren et al., 2016) to specifically look for variants with a predicted HIGH and MODERATE impact on the encoding proteins which will include non-synonymous changes. A total of 55,269 variants spanning 108 genes were analysed from 36 regions (**Table 3**). As expected, most of these variants were present in intronic (45.8%), intergenic (36.2%) and untranslated regions (1.2%), while a very small proportion can be considered true non-synonymous exonic variants (0.6%) (**Figure 4**). HIGH impact variants (n = 5) included variants at splice-donor/acceptor sites (n = 2) or which result in stop gain (n = 2) or start loss (n = 1), while 348 variants were predicted with MODERATE impact resulting from missense variation. Moreover, 71 of these variants had deleterious effects according to SIFT score. Overall, around 1/5 of variants (n = 75) were novel (without rsID) with a predicted HIGH or MODERATE impact.

**Table 3:** Details of variants from the WGS data in the candidate regions.

Genera	Number of variants from WGS data	Number of genes (protein-coding genes)	Number of deleterious variants (including low confidence)	Number of MODERATE OR HIGH impact variants (genes)	Genes detected in the regions
Campylobacter	14070	32 (25)	17	77 (18)	*UBE3C, DNAJB6, UFSP2, LRP2BP, SNX25, HOOK1*, *ENSGALG00000054726, ENSGALG00000050045, CYP2J23*, *XYLT1, SH2D2A, PRCC, METTL25B, ISG20L2, LOC425431*, *BCAN, HAPLN2, RHBG*
Cloacibacillus	13808	22 (14)	8	58 (10)	*SLC3A1, PREPL, CAMKMT, ST14, ADAMTS8, NSF, MEIOC*, *CCDC43, DBF4B, ADAM11*
Eisenbergiella	3648	7 (5)	1	5 (4)	*GRIN2A, CABIN1, TBX6, KLHL22*
Enterococcus	4986	9 (2)	11	28 (5)	*ENSGALG00000048821, ENSGALG00000052411*, *GRM7, ENSGALG00000048988, ENSGALG00000040705*
*Escherichia.Shigella*	2468	10 (7)	5	35 (3)	*AGO4, CLSPN, C1orf216*
*Helicobacter*	4595	9 (8)	27	107 (8)	*PTHLH, SPATS2L, KCTD18, SGO2, AOX1, CTGFL, EPB41*, *TMEM200B*
Parasutterella	4731	5 (4)	2	24 (3)	*CHORDC1, NAALAD2, FOLH1*
Sutterella	1956	5 (5)	0	4 (2)	*ENSGALG00000048789, GJA3*
Enterotype	5007	9 (9)	2	20 (6)	*AK7, PAPOLB, VRK1, C1QB, C1QA, EPHA8*

Candidate regions are defined by 50 kb upstream/downstream regions of significant GWAS associations.

Linear regression analysis revealed that out of 282 tested variants, 149 showed significant association (F-test BH corrected p < 0.05) with the respective phenotype (**Table 4**). This included 87 variants associated with *Helicobacter*, 47 variants with *Cloacibacillus*, 8 variants with Enterotype, 3 variants with *Sutterella*, and 2 variants each with *Campylobacter* and *Eisenbergiella*. The significant associations spanned 28 genes across all phenotypes. The higher number of variants associated with *Helicobacter* and *Cloacibacillus* were observed to have high LD among each other (**Supplementary Figures S13**–**S16**) potentially corresponding to the same haplotype.

**Table 4:** Variants from WGS data showing significant (BH adjusted p-value < 0.05) associations with respective trait/genera.

Trait/genera	SNP^a^	p-value	BH corrected p-value	Gene	Gene Symbol	Existing variation	Amino acid changes
Campylobacter	25_2669833_C/T	0.0091	0.0205	ENSGALG00000045469	BCAN	rs313794230	P/L
Campylobacter	25_2672688_C/T	0.0091	0.0205	ENSGALG00000013234	HAPLN2	rs734604429	R/H
Cloacibacillus	3_25589082_T/G	0.0046	0.0129	ENSGALG00000009973	SLC3A1		D/E
Cloacibacillus	3_25605238_C/T	0.0046	0.0129	ENSGALG00000009981	PREPL		R/Q
Cloacibacillus	3_25605251_T/C	0.0072	0.0175	ENSGALG00000009981	PREPL	rs16240366	T/A
Cloacibacillus	3_25605305_A/G	0.0072	0.0175	ENSGALG00000009981	PREPL	rs317010384	C/R
Cloacibacillus	3_25606137_C/T	0.0124	0.0261	ENSGALG00000009981	PREPL	rs313991750	E/K
Cloacibacillus	3_25606433_G/A	0.0057	0.0147	ENSGALG00000009981	PREPL	rs731329903	A/V
Cloacibacillus	3_25606528_A/T	0.0004	0.0014	ENSGALG00000009981	PREPL	rs316589833	H/Q
Cloacibacillus	3_25606546_G/C	0.0046	0.0129	ENSGALG00000009981	PREPL	rs312958834	H/Q
Cloacibacillus	3_25611805_G/A	0.0129	0.0269	ENSGALG00000009981	PREPL	rs317078462	R/C
Cloacibacillus	3_25616880_T/C	0.0057	0.0147	ENSGALG00000009981	PREPL	rs14329070	I/M
Cloacibacillus	3_25618988_G/A	0.0114	0.0245	ENSGALG00000009981	PREPL	rs312463147	P/L
Cloacibacillus	3_25619078_T/C	0.0114	0.0245	ENSGALG00000009981	PREPL	rs732499220	H/R
Cloacibacillus	3_25620636_G/A	0.0046	0.0129	ENSGALG00000035800	CAMKMT		G/S
Cloacibacillus	3_25624531_A/G	0.0121	0.0257	ENSGALG00000035800	CAMKMT	rs317444190	I/V
Cloacibacillus	24_1611093_A/G	0.0034	0.0099	ENSGALG00000001331	ST14	rs312708670	K/R
Cloacibacillus	24_1612920_G/A	0.0075	0.0177	ENSGALG00000001331	ST14	rs1059426179	V/I
Cloacibacillus	24_1687884_C/T	0.0007	0.0021	ENSGALG00000001370	ADAMTS8	rs13604433	V/M
Cloacibacillus	24_1688084_C/G	0.0069	0.0174	ENSGALG00000001370	ADAMTS8		G/A
Cloacibacillus	24_1688135_T/G	0.0018	0.0054	ENSGALG00000001370	ADAMTS8	rs315837753	K/T
Cloacibacillus	27_3610195_G/T	0.0016	0.0050	ENSGALG00000045889	MEIOC	rs315286505	A/S
Cloacibacillus	27_3612103_C/G	0.0016	0.0050	ENSGALG00000045889	MEIOC	rs315588936	D/E
Cloacibacillus	27_3612228_T/C	0.0052	0.0137	ENSGALG00000045889	MEIOC	rs314843968	V/A
Cloacibacillus	27_3612372_G/A	0.0029	0.0085	ENSGALG00000045889	MEIOC	rs318122260	S/N
Cloacibacillus	27_3612887_G/A	0.0029	0.0085	ENSGALG00000045889	MEIOC	rs313147053	G/S
Cloacibacillus	27_3613232_A/G	0.0096	0.0216	ENSGALG00000045889	MEIOC	rs317340940	S/G
Cloacibacillus	27_3613350_A/C	0.0111	0.0244	ENSGALG00000045889	MEIOC	rs318153894	N/T
Cloacibacillus	27_3627316_C/T	0.0052	0.0137	ENSGALG00000001022	CCDC43		D/N
Cloacibacillus	27_3639248_T/C	0.0070	0.0174	ENSGALG00000035814		rs738917549	L/P
Cloacibacillus	27_3639482_A/T	0.0049	0.0135	ENSGALG00000035814		rs738986282	S/C
Cloacibacillus	27_3640321_G/A	0.0075	0.0177	ENSGALG00000035814		rs317977273	R/H
Cloacibacillus	27_3640350_A/C	0.0065	0.0166	ENSGALG00000035814		rs732723155	T/P
Cloacibacillus	27_3640608_C/T	0.0071	0.0175	ENSGALG00000035814		rs739102177	A/V
Cloacibacillus	27_3640826_A/C	0.0156	0.0314	ENSGALG00000035814		rs733982035	T/P
Cloacibacillus	27_3640836_T/C	0.0133	0.0274	ENSGALG00000035814		rs741616962	V/A
Cloacibacillus	27_3640904_G/A	0.0227	0.0445	ENSGALG00000035814		rs738750878	G/R
Cloacibacillus	27_3641084_G/A	0.0114	0.0245	ENSGALG00000035814		rs739858911	V/M
Cloacibacillus	27_3641126_C/T	0.0054	0.0142	ENSGALG00000035814		rs735080961	R/W
Cloacibacillus	27_3641677_T/G	0.0075	0.0177	ENSGALG00000035673	ADAM11		W/G
Cloacibacillus	27_3641732_G/A	0.0012	0.0039	ENSGALG00000035673	ADAM11	rs313453668	R/Q
Cloacibacillus	27_3641847_C/T	0.0052	0.0137	ENSGALG00000035673	ADAM11	rs1060067070	R/C
Cloacibacillus	27_3641860_T/C	0.0133	0.0274	ENSGALG00000035673	ADAM11	rs314790078	I/T
Cloacibacillus	27_3642091_A/G	0.0139	0.0282	ENSGALG00000035673	ADAM11		E/G
Cloacibacillus	27_3642096_C/G	0.0085	0.0198	ENSGALG00000035673	ADAM11		R/G
Cloacibacillus	27_3642279_C/T	0.0114	0.0245	ENSGALG00000035673	ADAM11		P/S
Cloacibacillus	27_3644302_G/A	0.0090	0.0205	ENSGALG00000035673	ADAM11	rs737876624	G/S
Cloacibacillus	27_3645349_C/A	0.0052	0.0137	ENSGALG00000035673	ADAM11	rs1057892493	L/I
Cloacibacillus	27_3645523_G/A	0.0074	0.0177	ENSGALG00000035673	ADAM11	rs1058886917	R/Q
Eisenbergiella	14_9800969_G/A	0.0043	0.0125	ENSGALG00000007278	GRIN2A	rs316241099	
Eisenbergiella	15_8535646_G/C	0.0045	0.0129	ENSGALG00000006374	TBX6	rs14092889	S/T
Enterotype	5_46665025_T/C	0.0219	0.0431	ENSGALG00000011093	AK7	rs737231517	F/L
Enterotype	5_46705202_A/G	0.0237	0.0461	ENSGALG00000033099	PAPOLB	rs740228499	N/S
Enterotype	5_46806229_T/G	0.0110	0.0244	ENSGALG00000011116	VRK1	rs316371207	I/R
Enterotype	5_46809527_A/G	0.0251	0.0484	ENSGALG00000011116	VRK1	rs317850778	M/V
Enterotype	21_6073737_T/C	0.0260	0.0491	ENSGALG00000004771	C1QB		M/V
Enterotype	21_6073941_C/T	0.0260	0.0491	ENSGALG00000004771	C1QB		V/I
Enterotype	21_6074132_G/A	0.0260	0.0491	ENSGALG00000004771	C1QB		P/L
Enterotype	21_6119830_G/A	0.0194	0.0386	ENSGALG00000021567	EPHA8		T/M
Helicobacter	1_72755936_T/C	0.0001	0.0003	ENSGALG00000017295	PTHLH	rs13997156	Y/C
Helicobacter	7_11147457_T/A	0.0002	0.0006	ENSGALG00000008152	SPATS2L	rs316608554	C/S
Helicobacter	7_11149713_A/G	0.0002	0.0006	ENSGALG00000008152	SPATS2L	rs740737513	N/S
Helicobacter	7_11175092_T/A	0.0001	0.0003	ENSGALG00000008155	KCTD18	rs315734161	E/D
Helicobacter	7_11175142_T/C	0.0001	0.0006	ENSGALG00000008155	KCTD18	rs740199772	K/E
Helicobacter	7_11175328_G/T	1.19E-05	0.0003	ENSGALG00000008155	KCTD18	rs315522429	R/S
Helicobacter	7_11175453_G/A	1.19E-05	0.0003	ENSGALG00000008155	KCTD18	rs736999343	S/L
Helicobacter	7_11184247_C/A	0.0003	0.0009	ENSGALG00000045898		rs16583427	P/T
Helicobacter	7_11184254_C/A	0.0001	0.0004	ENSGALG00000045898		rs16583428	A/D
Helicobacter	7_11184266_C/T	0.0001	0.0003	ENSGALG00000045898		rs14606992	P/L
Helicobacter	7_11184269_C/T	0.0002	0.0008	ENSGALG00000045898		rs16583429	P/L
Helicobacter	7_11186981_C/T	2.10E-05	0.0003	ENSGALG00000045898		rs316081641	A/V
Helicobacter	7_11187944_C/T	3.60E-05	0.0003	ENSGALG00000045898		rs314367404	P/S
Helicobacter	7_11188145_G/A	0.0002	0.0006	ENSGALG00000045898		rs16583439	V/I
Helicobacter	7_11188398_T/C	8.75E-06	0.0003	ENSGALG00000045898		rs314125300	V/A
Helicobacter	7_11188446_A/G	8.75E-06	0.0003	ENSGALG00000045898		rs315014497	N/S
Helicobacter	7_11188577_C/G	0.0001	0.0003	ENSGALG00000045898		rs317892365	L/V
Helicobacter	7_11188625_T/C	0.0001	0.0003	ENSGALG00000045898		rs313958299	Y/H
Helicobacter	7_11188656_A/G	0.0001	0.0003	ENSGALG00000045898		rs734152116	E/G
Helicobacter	7_11188659_G/A	0.0001	0.0003	ENSGALG00000045898		rs738961881	S/N
Helicobacter	7_11188674_T/C	0.0001	0.0003	ENSGALG00000045898		rs736320705	L/S
Helicobacter	7_11188683_T/C	0.0001	0.0003	ENSGALG00000045898		rs738401633	V/A
Helicobacter	7_11188713_G/A	0.0001	0.0003	ENSGALG00000045898		rs312743552	S/N
Helicobacter	7_11188722_A/G	0.0001	0.0003	ENSGALG00000045898		rs312312117	N/S
Helicobacter	7_11188890_A/C	0.0001	0.0003	ENSGALG00000045898		rs315716018	K/T
Helicobacter	7_11188939_C/G	0.0001	0.0003	ENSGALG00000045898		rs315229324	N/K
Helicobacter	7_11188992_A/T	3.60E-05	0.0003	ENSGALG00000045898		rs316877223	N/I
Helicobacter	7_11188999_A/G	3.60E-05	0.0003	ENSGALG00000045898		rs732721600	I/M
Helicobacter	7_11189090_A/G	8.75E-06	0.0003	ENSGALG00000045898		rs316141263	S/G
Helicobacter	7_11189121_C/G	0.0001	0.0003	ENSGALG00000045898		rs317637097	P/R
Helicobacter	7_11189168_G/T	1.10E-05	0.0003	ENSGALG00000045898		rs737345480	D/Y
Helicobacter	7_11189316_C/T	9.21E-06	0.0003	ENSGALG00000045898		rs317012808	S/L
Helicobacter	7_11189562_T/C	4.15E-05	0.0003	ENSGALG00000045898		rs317439407	F/S
Helicobacter	7_11189660_A/T	3.58E-05	0.0003	ENSGALG00000045898		rs318185039	I/F
Helicobacter	7_11189685_G/A	0.0001	0.0003	ENSGALG00000045898		rs316960167	R/K
Helicobacter	7_11189756_A/G	3.16E-05	0.0003	ENSGALG00000045898		rs734098887	I/V
Helicobacter	7_11189757_T/C	3.33E-05	0.0003	ENSGALG00000045898		rs736755810	I/T
Helicobacter	7_11189840_T/A	3.58E-05	0.0003	ENSGALG00000045898		rs14607000	S/T
Helicobacter	7_11191962_C/T	3.60E-05	0.0003	ENSGALG00000045898		rs316014568	S/F
Helicobacter	7_11201136_C/G	0.0001	0.0003	ENSGALG00000008185	AOX1	rs735036820	S/C
Helicobacter	7_11201777_C/A	0.0001	0.0006	ENSGALG00000008185	AOX1	rs740659172	T/N
Helicobacter	7_11206191_C/A	0.0002	0.0006	ENSGALG00000008185	AOX1	rs16583520	D/E
Helicobacter	7_11206457_C/T	0.0002	0.0008	ENSGALG00000008185	AOX1	rs317893303	R/C
Helicobacter	7_11208224_G/A	2.98E-05	0.0003	ENSGALG00000008185	AOX1	rs316077433	V/I
Helicobacter	7_11214194_C/T	4.55E-05	0.0003	ENSGALG00000008185	AOX1		P/S
Helicobacter	7_11217194_C/G	4.62E-05	0.0003	ENSGALG00000008185	AOX1	rs733756210	Q/E
Helicobacter	7_11217213_G/A	0.0001	0.0005	ENSGALG00000008185	AOX1	rs312867661	R/K
Helicobacter	7_11218059_A/G	0.0002	0.0006	ENSGALG00000008185	AOX1	rs732046936	K/R
Helicobacter	7_11220766_A/G	0.0001	0.0003	ENSGALG00000008185	AOX1	rs313890332	T/A
Helicobacter	7_11220787_A/G	0.0001	0.0006	ENSGALG00000008185	AOX1		I/V
Helicobacter	7_11220838_A/G	0.0002	0.0006	ENSGALG00000008185	AOX1	rs314774615	I/V
Helicobacter	7_11221826_A/G	0.0002	0.0006	ENSGALG00000008185	AOX1	rs16583544	K/R
Helicobacter	7_11222992_A/G	0.0002	0.0006	ENSGALG00000008185	AOX1	rs733522999	T/A
Helicobacter	7_11223404_C/T	0.0001	0.0003	ENSGALG00000008185	AOX1	rs738494931	R/C
Helicobacter	23_2738339_A/G	0.0001	0.0006	ENSGALG00000034756		rs739929788	K/R
Helicobacter	23_2740678_G/A	3.67E-05	0.0003	ENSGALG00000034756		rs317304529	E/K
Helicobacter	23_2741265_C/T	4.18E-05	0.0003	ENSGALG00000034756		rs316958545	R/C
Helicobacter	23_2741529_A/G	4.56E-05	0.0003	ENSGALG00000034756		rs736685773	T/A
Helicobacter	23_2743115_G/T	0.0001	0.0004	ENSGALG00000001329	EPB41	rs737893868	E/*
Helicobacter	23_2743116_A/T	0.0001	0.0004	ENSGALG00000001329	EPB41	rs731614152	E/V
Helicobacter	23_2743121_C/A	0.0002	0.0006	ENSGALG00000001329	EPB41	rs1059519464	H/N
Helicobacter	23_2743133_C/A	0.0001	0.0006	ENSGALG00000001329	EPB41	rs735026334	R/S
Helicobacter	23_2743197_G/C	0.0001	0.0005	ENSGALG00000001329	EPB41	rs1059721572	G/A
Helicobacter	23_2743352_C/G	5.81E-06	0.0003	ENSGALG00000001329	EPB41		L/V
Helicobacter	23_2743386_G/C	0.0001	0.0004	ENSGALG00000001329	EPB41		R/P
Helicobacter	23_2743388_C/T	0.0001	0.0004	ENSGALG00000001329	EPB41		P/S
Helicobacter	23_2743472_G/A	8.08E-06	0.0003	ENSGALG00000001329	EPB41		G/S
Helicobacter	23_2743572_T/C	4.87E-05	0.0003	ENSGALG00000001329	EPB41		L/P
Helicobacter	23_2743673_C/T	4.87E-05	0.0003	ENSGALG00000001329	EPB41		R/W
Helicobacter	23_2743694_G/A	0.0003	0.0010	ENSGALG00000001329	EPB41	rs1058972837	G/S
Helicobacter	23_2776735_C/T	1.41E-05	0.0003	ENSGALG00000001329	EPB41	rs313348793	A/V
Helicobacter	23_2792624_C/T	0.0002	0.0006	ENSGALG00000001329	EPB41	rs314369122	A/V
Helicobacter	23_2810511_G/A	0.0001	0.0003	ENSGALG00000001329	EPB41		R/H
Helicobacter	23_2810580_C/T	0.0002	0.0006	ENSGALG00000001329	EPB41	rs315620767	A/V
Helicobacter	23_2810625_C/T	1.49E-06	0.0003	ENSGALG00000001329	EPB41	rs739930131	A/V
Helicobacter	23_2810660_G/A	0.0002	0.0006	ENSGALG00000001329	EPB41	rs733380008	E/K
Helicobacter	23_2810662_A/T	0.0001	0.0006	ENSGALG00000001329	EPB41	rs314995393	E/D
Helicobacter	23_2823181_T/C	0.0001	0.0003	ENSGALG00000024318	TMEM200B	rs316289949	S/G
Helicobacter	23_2823275_G/T	0.0001	0.0004	ENSGALG00000024318	TMEM200B	rs735947939	S/R
Helicobacter	23_2823502_A/C	1.83E-05	0.0003	ENSGALG00000024318	TMEM200B	rs733254337	Y/D
Helicobacter	23_2823570_C/T	0.0002	0.0006	ENSGALG00000024318	TMEM200B	rs312912847	G/D
Helicobacter	23_2824111_G/A	1.87E-05	0.0003	ENSGALG00000024318	TMEM200B	rs316556149	R/*
Helicobacter	23_2824129_T/C	0.0001	0.0003	ENSGALG00000024318	TMEM200B	rs315330603	T/A
Helicobacter	23_2824224_C/T	0.0001	0.0003	ENSGALG00000024318	TMEM200B	rs14289505	S/N
Helicobacter	23_2824285_G/A	0.0001	0.0006	ENSGALG00000024318	TMEM200B	rs733720106	L/F
Helicobacter	23_2824359_C/T	0.0001	0.0004	ENSGALG00000024318	TMEM200B	rs316435782	R/H
Helicobacter	23_2824360_G/A	0.0002	0.0007	ENSGALG00000024318	TMEM200B	rs14289507	R/C
Sutterella	1_180296047_G/A	0.0138	0.0282	ENSGALG00000048789		rs316343115	L/F
Sutterella	1_180296227_G/A	0.0086	0.0200	ENSGALG00000048789		rs15522683	R/C
Sutterella	1_180313529_A/G	0.0160	0.0319	ENSGALG00000017137	GJA3	rs315165625	S/G

a SNP is represented by chromosome, position, reference allele/alternate allele separated by ‘_’.

## 4 Discussion

We have investigated the relationship between host genetic variation and the abundance or presence of specific microbial genera within the chicken caecal microbiota, providing insights into host-microbiome interactions.

We observed that 6 out of 10 microbial genera exhibited non-zero heritability estimates, indicating that while some microbial genera show a measurable genetic component, others do not. This is in line with previous studies, which also demonstrated that genetic variation in abundance or occurrence of microbes is not present for all (Goodrich et al., 2016a; Feng et al., 2022). Interestingly, studies comparing human and mouse microbiomes have reported higher heritability estimates in mice, which were sampled in controlled laboratory settings (Goodrich et al., 2016b). Similar factors could impact the current study as samples were collected from natural settings such as poultry farms. Despite these limitations, heritability trends were consistent with relevant prior field studies. For instance, our study showed a significant heritability of 0.2 for *Campylobacter* which is comparable with previous studies (0.11 and 0.25) (Psifidi et al., 2016b; Psifidi et al., 2021), while similar levels of heritability were also observed for *Escherichia* and *Lactobacillus* (Mignon-Grasteau et al., 2015). The low heritability of some microbial taxa could also stem from the inability of current short-read sequencing technologies to reliably achieve species-level resolution (Deng et al., 2021). This limitation hinders reliable heritability estimates and complicates cross-study comparisons, as true genetic influences are obscured by methodological inconsistencies.

GWAS analysis across bird ecotypes revealed multiple novel genetic associations with the abundance of different genera. However, we didn’t observe any overlaps in candidate regions between across-ecotype (current study) GWAS and within-ecotype-GWAS (Dai et al., 2022) even though both displayed similar levels of heritability. These findings suggest that while genetic influence on microbiome composition may be consistent across ecotypes, the specific genetic architecture of such traits may vary significantly. In addition, the present study demonstrated the usefulness of joint analysis, since the bigger sample size made it possible to reveal further genetic associations common between different chicken ecotypes. This is in accordance with previous chicken genetic studies (Banos et al., 2020). While multiple associations were observed on the same chromosomes, we did not observe any overlapping candidate regions between different genera/studied traits. *Campylobacter* and *Cloacibacillus* showed the highest number (n = 9) of significant SNP-genera associations. Interestingly, *Cloacibacillus* exhibited low heritability (h² = 0.084), emphasising that environmental factors play a much more important role than genetic ones. Such findings align with prior microbiome GWAS studies, which frequently report significant associations despite low heritability estimates (Rothschild et al., 2018; Grieneisen et al., 2021). The significant associations with *Campylobacter* were distributed over GGC2-4, GGC8, GGC10, GGC11, GGC14, and GGC25. However, this study did not identify any of the previously reported associations with *Campylobacter* in broiler chickens and the Barred Rock breed (Connell et al., 2013; Psifidi et al., 2021). Overall, a higher number of associations were observed on GGC1 (n = 9) across multiple genera. Of note, *Cloacibacillus* (5 out of 9) and *Parasutterella* (3 out of 5) had the most significant associations on GGC1.

Host-microbiome crosstalk is a complex, bidirectional process with multiple factors influencing them including immunological/inflammatory, regulatory/hormonal, signalling and metabolic processes (Sun et al., 2017; Zheng et al., 2020; Maciel-Fiuza et al., 2023). Several markers linked with candidate genes identified in this study are implicated in immune pathways and host-microbiome interactions. For instance, genes associated with *Campylobacter* abundance [cytochrome P450 family2 subfamily J (*CYP2J*), sorting nexin 25 (*SNX25*), and UFM1 specific peptidase 2 (*Ufsp2*)] are involved in immune regulation. *CYP2J* genes metabolise arachidonic acid, a precursor for inflammatory mediators in several immune-related disorders (Clarke et al., 2010). *SNX25* regulates TGF-β signalling, enhancing receptor degradation, and *Ufsp2* suppresses responses to interferon-γ (IFN-γ) and lipopolysaccharides (LPS), key microbial interaction molecules (Balce et al., 2021; Nishimura et al., 2021). A gene associated with *Eisenbergiella* abundance [T-box 6 (*TBX6*)] modulates Notch1 signalling, which is linked to bacterial infection responses (Yasuhiko et al., 2006; Gallenstein et al., 2023). Similarly, genes associated with *Cloacibacillus* abundance, such as calmodulin-lysine N-methyltransferase (*CAMKMT*), prolyl endopeptidase-like (*PREPL*), and solute carrier family 3 member 1(*SLC3A1*), have roles in immune response modulation and were implicated in high IFN-γ levels in cattle exposed to avian tuberculin purified protein derivative (Badia-Bringue et al., 2023). Moreover, WGS data analysis revealed specific candidate variants in *CAMKMT*, *PREPL*, *SLC3A1*, and *TBX6* genes that may be involved in genera abundance modulation.

Another noteworthy association was the potential involvement of the *C1qA*, *C1qB*, and *C1qC* genes in Enterotype shaping. C1q is the recognition component of the C1 complex which initiates the classical pathway of complement activation by binding with and sensing immunological complexes like antigen-antibody complex, and non-immunological components like bacterial LPS (Mellors et al., 2020). Various studies have highlighted the role of the C1q molecule against microbial infections, describing how C1q deficiency leads to increased host susceptibility to various bacterial infections, and conversely showing the over-expression of C1q genes post bacterial infection (Lu et al., 2008; Rychlik et al., 2014). Moreover, studies in humans have shown that mutations in C1q genes lead to deficiency or lower levels of C1q and are strongly associated with clinical presentation in the form of encapsulated bacterial infections and systemic lupus erythematosus (SLE) (van Schaarenburg et al., 2016). The significant association in the current study was observed to be downstream of the *C1qA* gene and we observed 3 novel variants in WGS analysis (GGC21:6,073,737; GGC21:6,073,941; and GGC21:6,074,132) in *C1qB* gene showing significant association with Enterotype. All these taken together point towards potential interactions among gut microbiome members, components of the host complement system, and efferocytosis mechanisms, the outcome of which could shape the overall microbiota structure in the caeca. Efferocytosis is a process by which phagocytic cells remove dead and apoptotic cells, and has been previously linked with the inflammatory response and inflammatory disorders (Ge et al., 2022). Previous studies have reported that the gut microbiome, through its diverse array of metabolites, exerts a regulatory influence on efferocytosis in peripheral tissues, facilitating immune resolution and tissue repair while maintaining systemic homeostasis, as evidenced by its impact on macrophage polarisation and apoptotic cell clearance in inflammatory contexts (Li et al., 2021; Saavedra et al., 2022; Gorreja et al., 2023; Traughber et al., 2024).

The candidate region for Enterotype association also harboured two Ephrin (Eph) receptor genes (*EPHA8* and *EPHB2*), while in the candidate region for *Parasutterella* abundance another Ephrin receptor gene *EPHA3* was located. Eph receptors are receptor tyrosinase kinases that are involved in transducing extracellular signals inside the cells through ligand-induced activation (Lisabeth et al., 2013). Several EPH receptors and their ligands are also involved in immunological processes. For instance, *EPHB2* mutations and expression have been linked with colorectal cancer, while *EPHA2* and *EPHA3* are shown to have roles as viral entry receptors for herpesvirus and Epstein-Barr virus (Genander et al., 2009; Darling and Lamb, 2019). Other studies also report that Eph receptors are manipulated by bacteria for immune evasion, while other studies have described their involvement in the activation of immune cells (Funk and Orr, 2013; Yu et al., 2014). Moreover, a significant association (GGC21:6,119,830) from WGS analysis with Enterotype trait further underscores the importance of these genes in host-microbiome interactions. However, further studies are needed to confirm this speculation and establish detailed interactions and mechanisms.

The current study also identified several other genes in the candidate regions of association which code for proteins found in the extracellular matrix (ECM) that interact with membrane receptors. Gut bacteria engage in intricate interactions with the ECM and produce a variety of enzymes and metabolites that can degrade ECM components, influencing tissue architecture and permeability (Derkacz et al., 2021). ECM components can serve as signalling molecules that affect bacterial behaviour, including biofilm formation and virulence. The ability of gut microbes to interact with the ECM also highlights the bidirectional communication between host tissues and the microbiota, which is crucial for maintaining gut homeostasis (Franchi et al., 2024). Notably, *BCAN* (brevican, associated with *Campylobacter*) and *HAPLN2* (hyaluronan and proteoglycan link protein 2, associated with *Campylobacter*) proteins involved in the binding of hyaluronic acid; G-protein coupled receptors *GRM7* (glutamate metabotropic receptor 7, associated with *Enterococcus* abundance) and *TACR3* (tachykinin receptor 3, associated with *Campylobacter*); glutamate receptor *GRIN2A* (associated with *Eisenbergiella*); heat-shock protein binding genes *CHORDC1* (cysteine and histidine rich domain containing 1, associated with *Parasutterella* abundance), *DNAJB6* (DnaJ heat shock protein family (Hsp40) member B6, associated with *Campylobacter* abundance), and *LOC425431* (dnaJ homolog subfamily A member 1-like, associated with *Campylobacter* abundance) were observed in the candidate regions. Of these, many genes have been previously shown to interact with bacteria and viruses in the gut. For instance, the *GRM7* gene was identified as a candidate gene in GWAS with antibody titres for *Salmonella enterica* serovar Gallinarum in chicken (Psifidi et al., 2016a). The *CHORDC1* gene was observed to be upregulated post-infection from Reovirus, Hepatitis B virus, and Newcastle disease virus in chickens (Walugembe et al., 2020; Fogel et al., 2021). Transcription of the gene *DNAJB6* was observed to be significantly upregulated by porcine circovirus type 2 infection in porcine cell lines (Han et al., 2020) and also observed to be significantly overexpressed in chicken cell lines after treatment with *Salmonella enterica* serovar Typhimurium derived lipopolysaccharide (Slawinska et al., 2016).

The current study identified associations between bacterial abundance or occurrence and genes involved in the metabolism of Retinol (Vitamin A1), Thiamine (Vitamin B1), Vitamin B6, Nicotinamide (Vitamin B3) and Folate. These micronutrients serve as essential cofactors for microbial metabolic pathways, influencing bacterial growth and survival. For instance, B vitamins, particularly B12 and folate, are critical for microbial metabolism and have been shown to impact the microbiota (Guetterman et al., 2022), while Vitamin A, through its active form, retinoic acid, affects gut immune responses, which in turn modulates the microbial environment (Pham et al., 2021). In our study, the *AOX1* (aldehyde oxidase 1) gene was observed in the candidate region for *Helicobacter* association. *AOX1* is involved in the oxidation of 9-cis and all-trans retinal (active form of Vitamin A) into the corresponding retinoic acid; conversion of 1-Methylnicotinamide (a primary metabolite of nicotinamide and shown to have roles in immune modulation of T cells in cancer) to 1-methyl-2-pyridone-5-carboxamide or 1-methyl-4-pyridone-5-carboxamide; and oxidation of Pyridoxal (the vitamin B6 precursor) to 4-pyridoxic acid (Terao et al., 2016; Kilgour et al., 2021). Another gene, *AK7* (adenylate kinase 7) (associated with Enterotype), is involved in the conversion of thiamine diphosphate, the most abundant physiological form of thiamine and vital coenzyme for enzymatic reactions, to thiamine triphosphate (Bettendorff, 2021). Both in *AK7* and *AOX1* we identified variants significantly associated with Enterotype and *Helicobacter*, respectively. *FOLH1* (folate hydrolase 1) and *NAALAD2* (N-acetylated alpha-linked acidic dipeptidase 2), which are involved in cellular transport and absorption specifically related to folate metabolism, were also observed to be associated with *Parasutterella* (belonging to proteobacterial phylum). Several Proteobacteria organisms in the gut are known to modulate folate bioavailability and in turn regulate the host expression of receptors (Rais et al., 2016; Engevik et al., 2019).

Interplay between host genetics and gut microbiota reflects a co-evolutionary process in which hosts and microbes have adapted to one another over time. While certain genetic variants are clearly associated with alterations in the gut microbiome, the precise mechanisms remain an area of active investigation, tackled by many across humans and animals/birds studies (Spor et al., 2011; Bonder et al., 2016; Kurilshikov et al., 2017). Genetic variation, particularly in immune-related genes, play a pivotal role in shaping the gut environment by promoting the growth of specific microbial communities while inhibiting others. However, the microbiome is highly breed-specific, as genetic differences may influence gut morphology, immune function, and metabolic processes, leading to distinct microbial compositions between breeds (Pandit et al., 2018; Cao et al., 2020; Paul et al., 2021; Davies et al., 2022). Additionally, environmental factors such as diet significantly influence the gut microbiota, making more challenging to dissect the genetic contribution (Spor et al., 2011). While birds were sampled from farms that provided standard poultry feed, diet-related microbiome variations can still arise due to differences in feed formulation, storage conditions, and microbial contamination (Benson et al., 2010). Moreover, geographical location further contributes to microbiota diversity, as climate, humidity, and altitude affect microbial colonisation and gut health (Kers et al., 2018). To reduce the noise, the farms sampled in this study were selected from a single state of India, thereby restricting to smaller geographic area. The selected farms were following similar managemental practices, and half of the selected farms reared both chicken lines simultaneously reducing the environmental noise. Moreover, we have incorporating environmental and farming variables into our statistical models, a strategy supported by previous research to reduce environmental noise in the analysis (Awany et al., 2018). Additionally, the use of high-dimensional genotyping arrays, while providing broad coverage, may still miss rare or structural variants contributing to microbiota variation, a limitation highlighted in GWAS studies on complex traits (Awany et al., 2018). Nevertheless, the interaction between the identified SNP-containing genes and symbiotic microorganisms provides crucial insights into host-microbiome relationships that can support breeding and selection strategies. Understanding how specific genetic variants modulate microbial composition and function can enable targeted breeding approaches that enhance beneficial microbial colonisation, indirectly improving disease resistance, nutrient utilisation, overall growth performance and reducing zoonotic burden. Future research integrating whole-genome sequencing and multi-omics approaches will be essential for a more comprehensive understanding of host-microbiota genetic interactions. By integrating microbiome-informed selection into genetic improvement programs, poultry breeding can move toward optimising gut health alongside other important health, production and sustainability traits.

## Conclusion

This study identifies candidate genes that provide a foundation for further exploration into the intricate interactions between host genetic polymorphisms and the chicken caecal microbiome. These findings offer valuable insights into how specific host genetic variants may modulate microbial composition and subsequently impact health outcomes. However, given the complex interplay between genetic and environmental factors including diet, these results must be interpreted cautiously and validated in larger populations under controlled conditions. Future studies integrating functional genomics, transcriptomics, and metabolomics will help establish causal relationships between host and the gut microbiota. Additionally, exploring host genetic-microbe interactions in multiple chicken breeds and production systems will help refine microbiome-based selection strategies and ensure their broader applicability in poultry breeding programs.

## Data Availability

The datasets presented in this study can be found in online repositories. The 16S rRNA gene sequence data is available from EBI-ENA (https://www.ebi.ac.uk/ena/browser/search) under Project ID PRJEB15343, SRA ID ERP017060.
