# Supplementary material for: Unveiling host-genetic drivers of caecal microbial communities in chickens through genome-wide association studies
Source: Front Microbiomes. 2025 Feb 18;4:1539923. doi: 10.3389/frmbi.2025.1539923 (PMC12993601; doi:10.3389/frmbi.2025.1539923)
Supplement: Supplementary file 1 [file DataSheet1.pdf]

Supplementary figures:

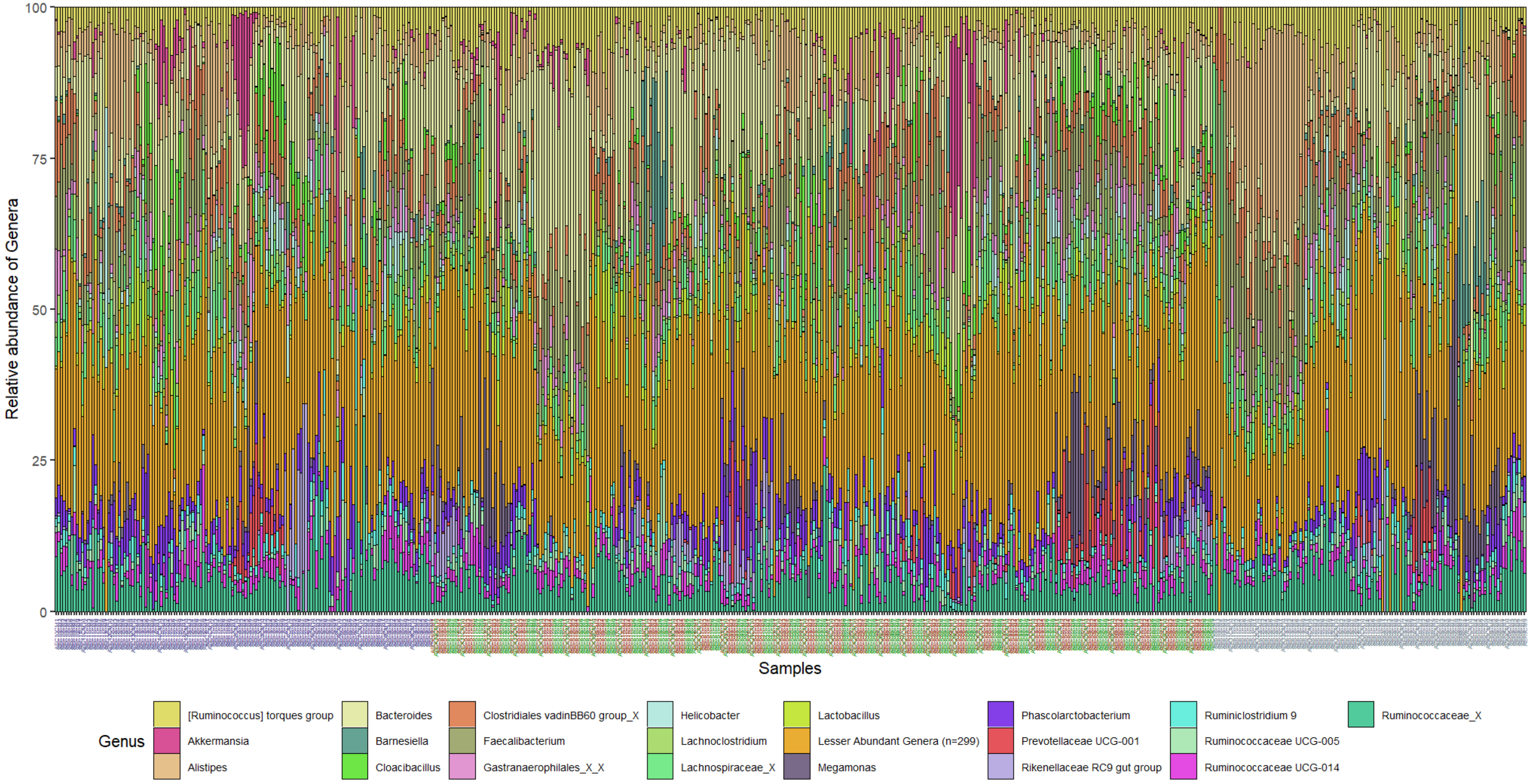

Figure S1: Genera-level abundance across all samples. Top abundant bacterial genera are represented individually, while 299 lesser abundant genera are pooled and represented together. The suffix '_X' is added to indicate unclassified taxa and multiple suffixes are used to indicate the last level of classification. The samples from broiler-only farms are labelled in blue, samples from Kadaknath-only farms are labelled in grey, while samples from mixed farms are labelled in red (Kadaknath) and green (broiler).

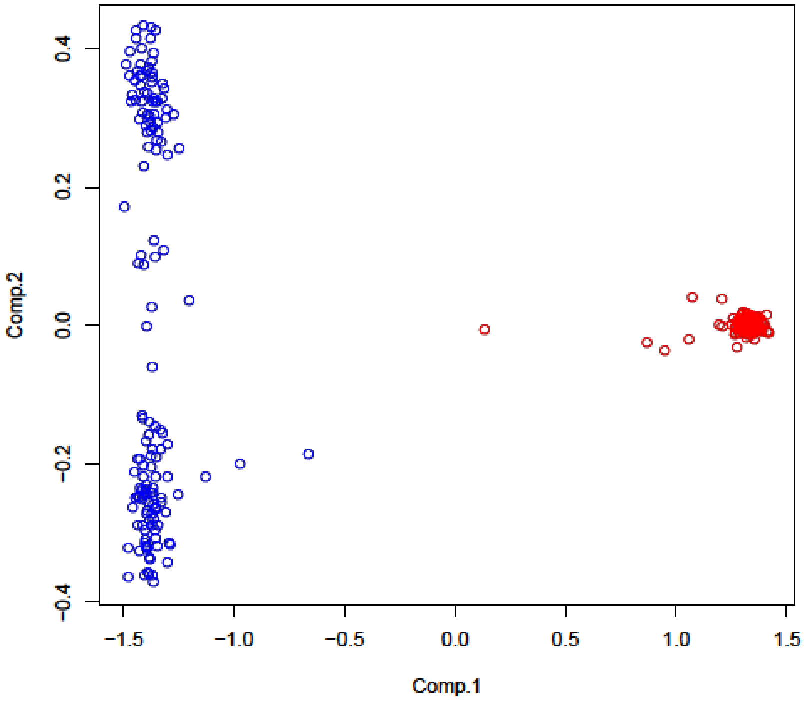

Figure S2: Multidimensional scaling analysis from genomic relationship matrix showing that Kadaknath (blue dots) and Cobb400 (red dots) are genetically distinct.

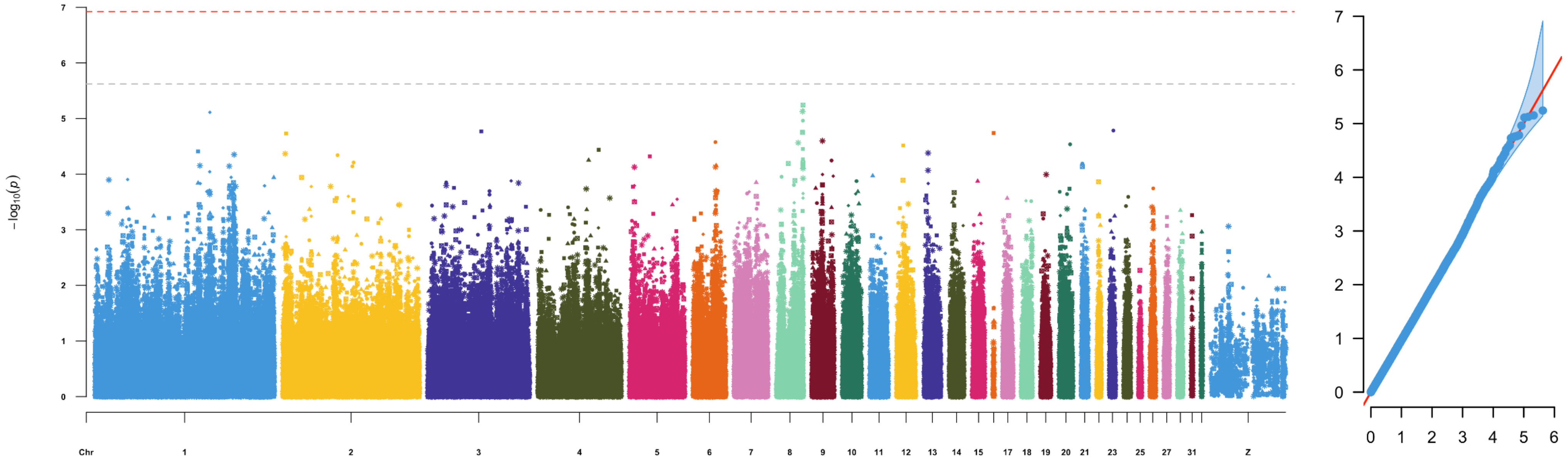

Figure S3: Manhattan plot (left) and QQplot (right) depicting -log$_{10}$ (p-value) from the GWAS results with genera *Bacillus*. The red line represents the genome-wide significance threshold, while the grey dash line represents the suggestive genome-wide threshold.

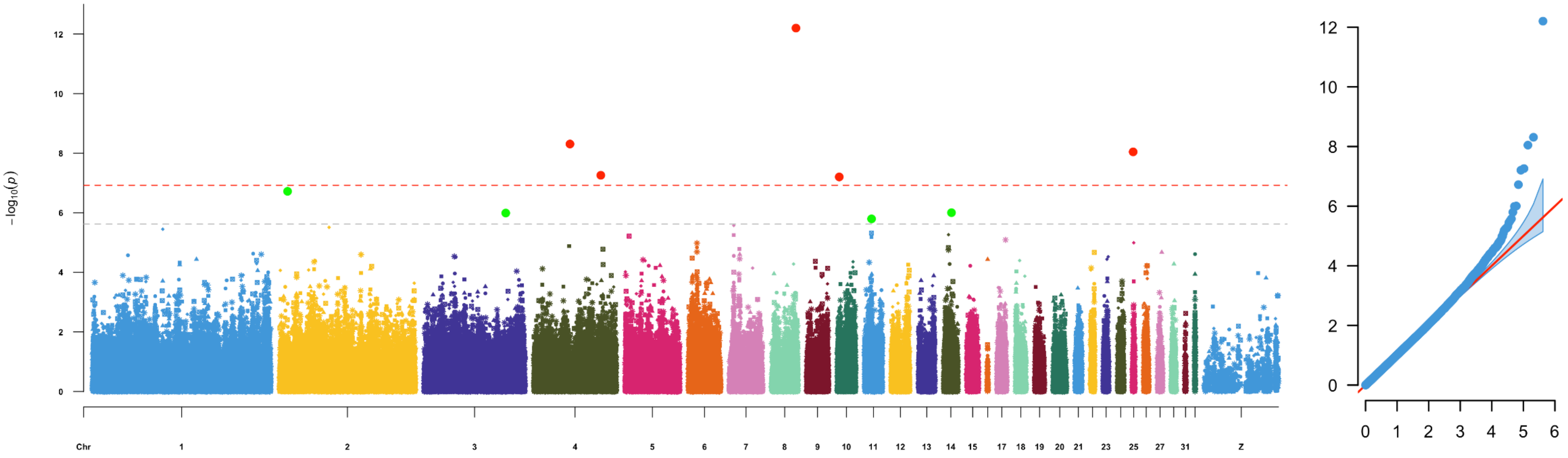

Figure S4: Manhattan plot (left) and QQplot (right) depicting -log_{10}(p-value) from the GWAS results with genera *Campylobacter*. The red line represents the genome-wide significance threshold, while the grey dash line represents the suggestive genome-wide threshold.

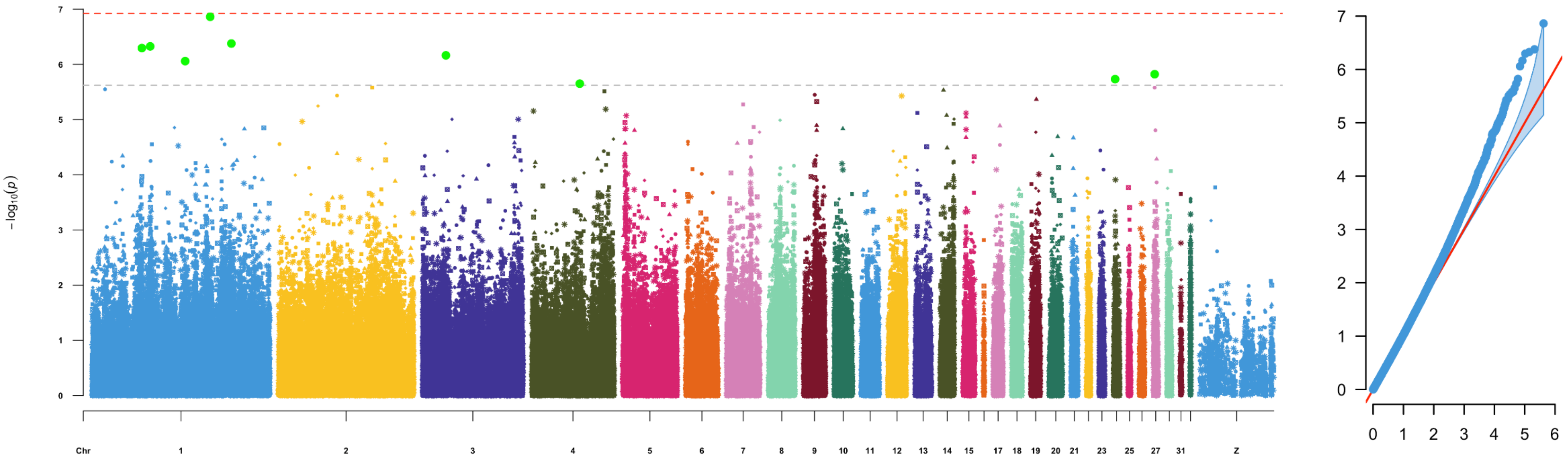

Figure S5: Manhattan plot (left) and QQplot (right) depicting -$\log_{10}$(p-value) from the GWAS results with genera *Cloacibacillus*. The red line represents the genome-wide significance threshold, while the grey dash line represents the suggestive genome-wide threshold.

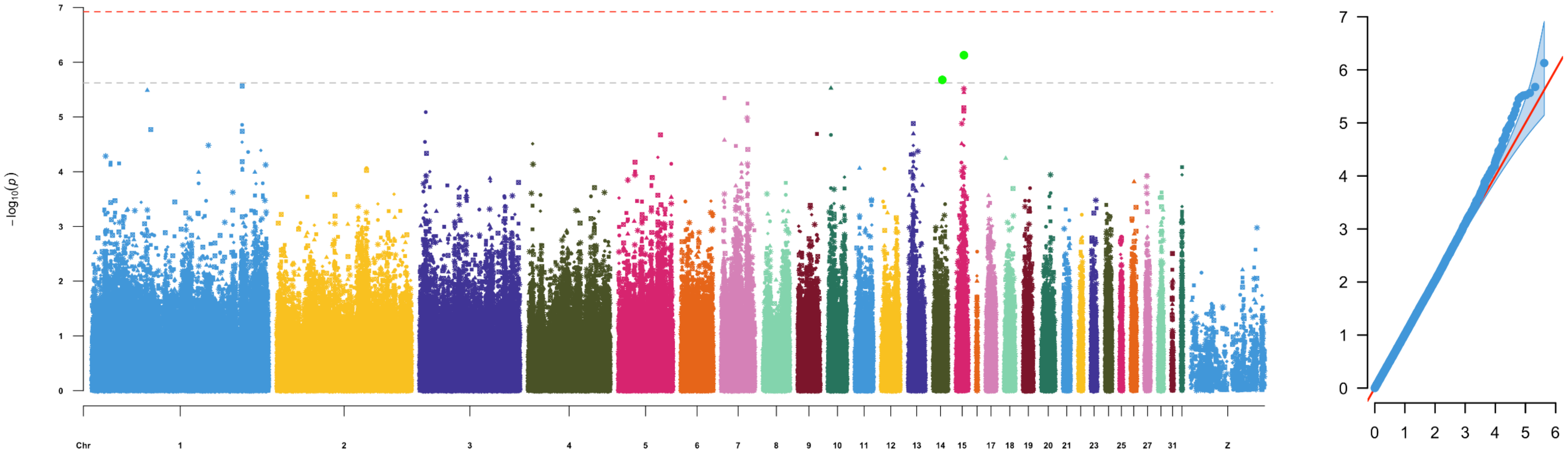

Figure S6: Manhattan plot (left) and QQplot (right) depicting -log$_{10}$(p-value) from the GWAS results with genera *Eisenbergiella*. The red line represents the genome-wide significance threshold, while the grey dash line represents the suggestive genome-wide threshold.

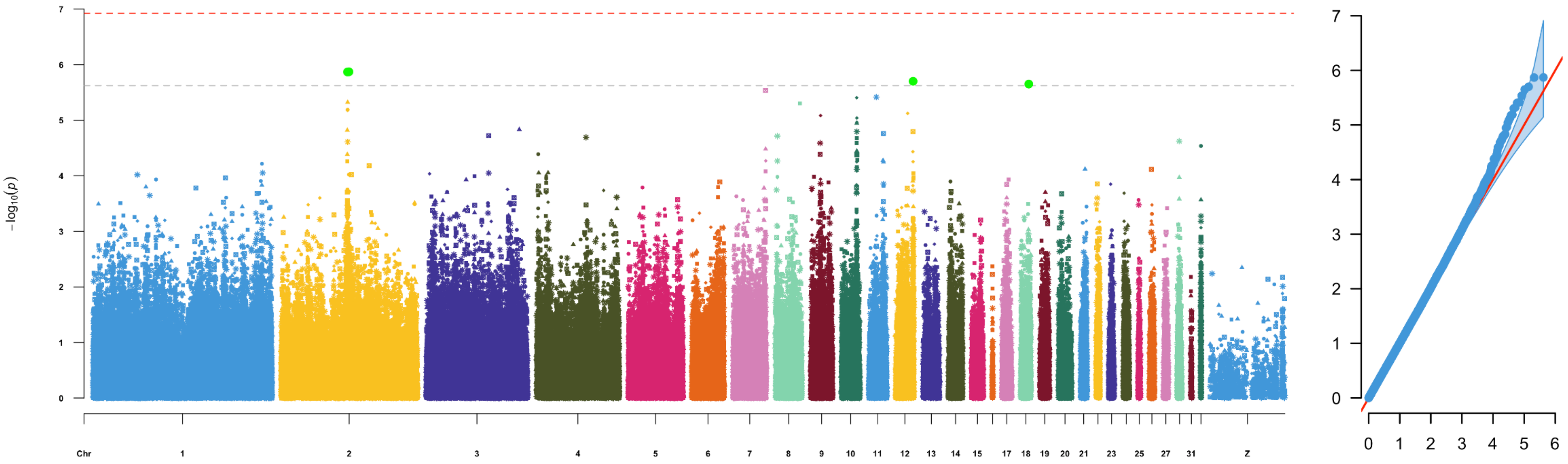

Figure S7: Manhattan plot (left) and QQplot (right) depicting -log$_{10}$(p-value) from the GWAS results with genera *Enterococcus*. The red line represents the genome-wide significance threshold, while the grey dash line represents the suggestive genome-wide threshold.

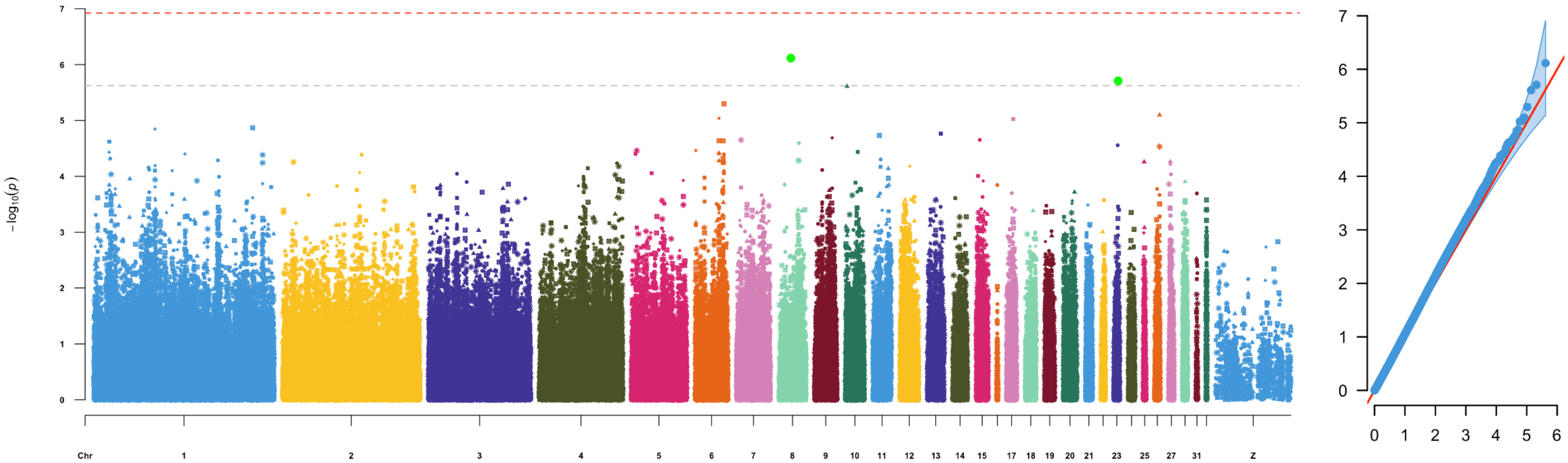

Figure S8: Manhattan plot (left) and QQplot (right) depicting $-\log_{10}$(p-value) from the GWAS results with genera *Escherichia.Shigella*. The red line represents the genome-wide significance threshold, while the grey dash line represents the suggestive genome-wide threshold.

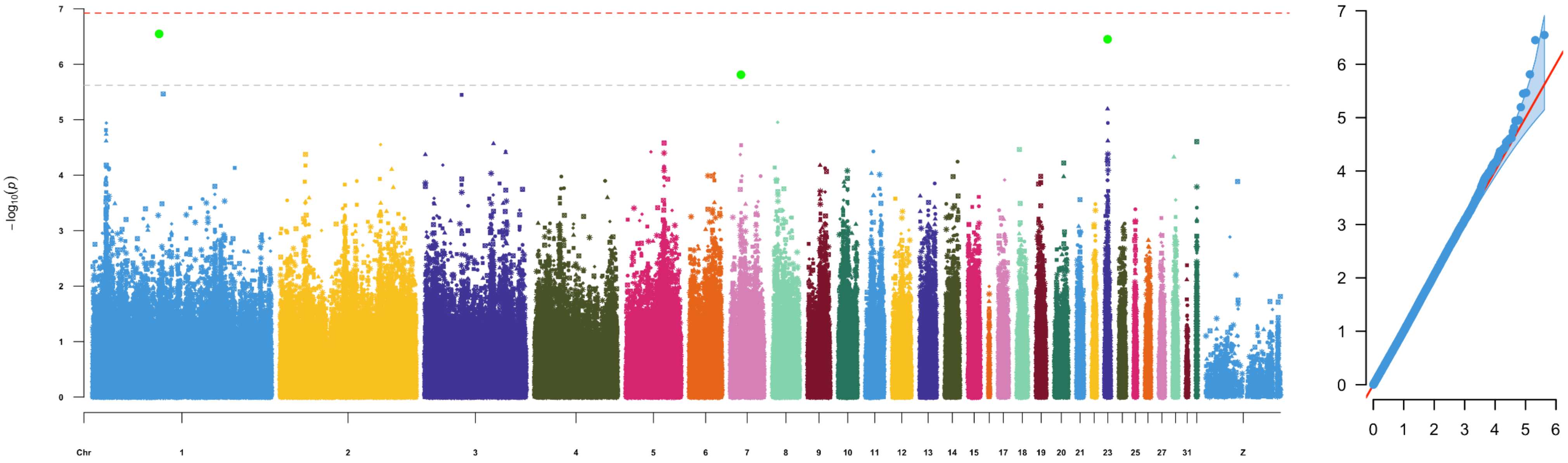

Figure S9: Manhattan plot (left) and QQplot (right) depicting -log$_{10}$(p-value) from the GWAS results with genera *Helicobacter*. The red line represents the genome-wide significance threshold, while the grey dash line represents the suggestive genome-wide threshold.

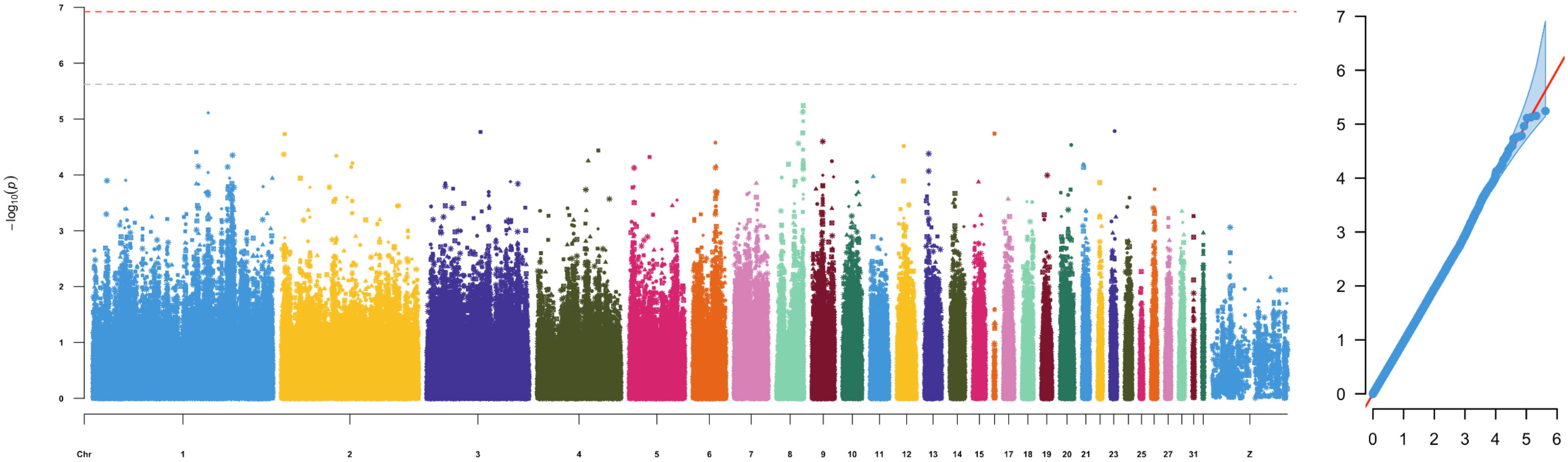

Figure S10: Manhattan plot (left) and QQplot (right) depicting -log$_{10}$(p-value) from the GWAS results with genera *Lactobacillus*. The red line represents the genome-wide significance threshold, while the grey dash line represents the suggestive genome-wide threshold.

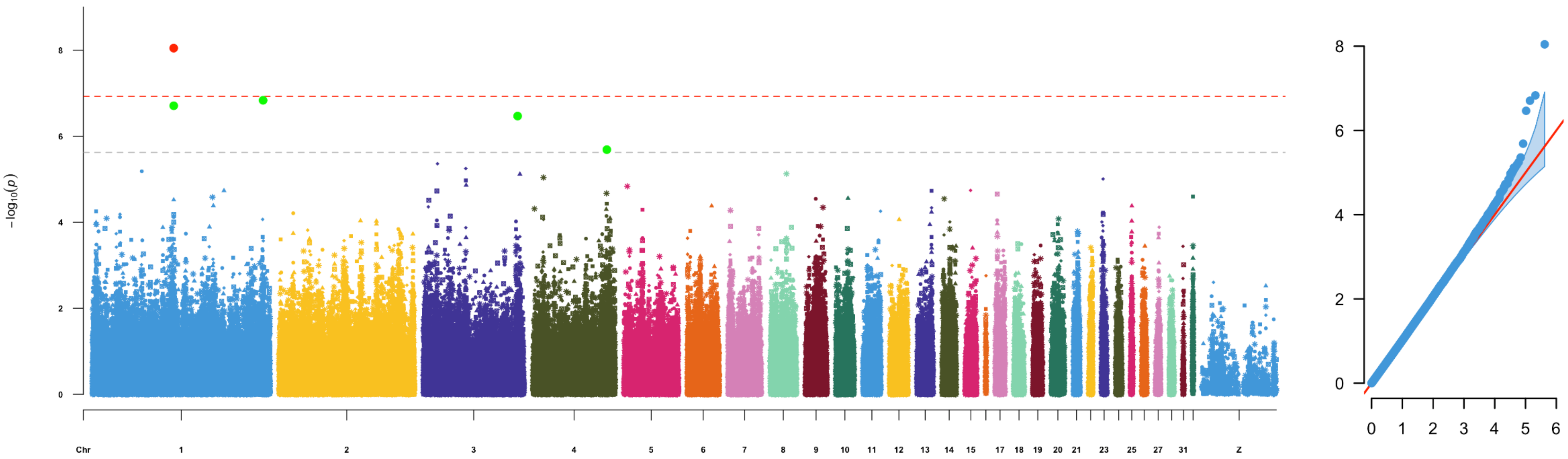

Figure S11: Manhattan plot (left) and QQplot (right) depicting -log$_{10}$(p-value) from the GWAS results with genera *Parasutterella*. The red line represents the genome-wide significance threshold, while the grey dash line represents the suggestive genome-wide threshold.

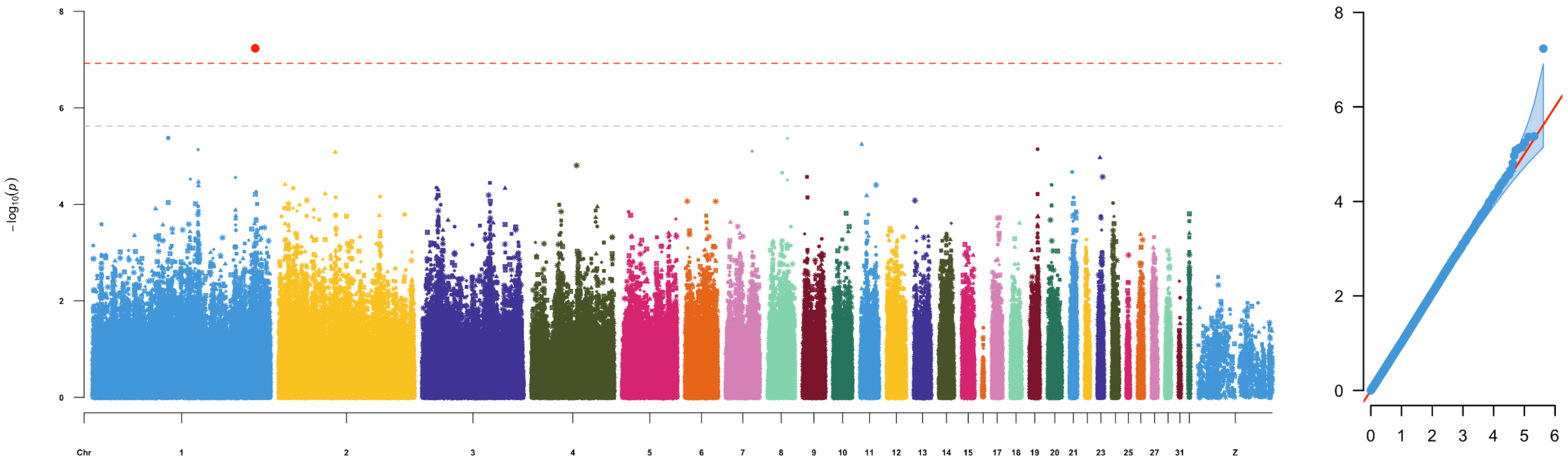

Figure S12: Manhattan plot (left) and QQplot (right) depicting -$\log_{10}$(p-value) from the GWAS results with genera *Sutterella*. The red line represents the genome-wide significance threshold, while the grey dash line represents the suggestive genome-wide threshold.

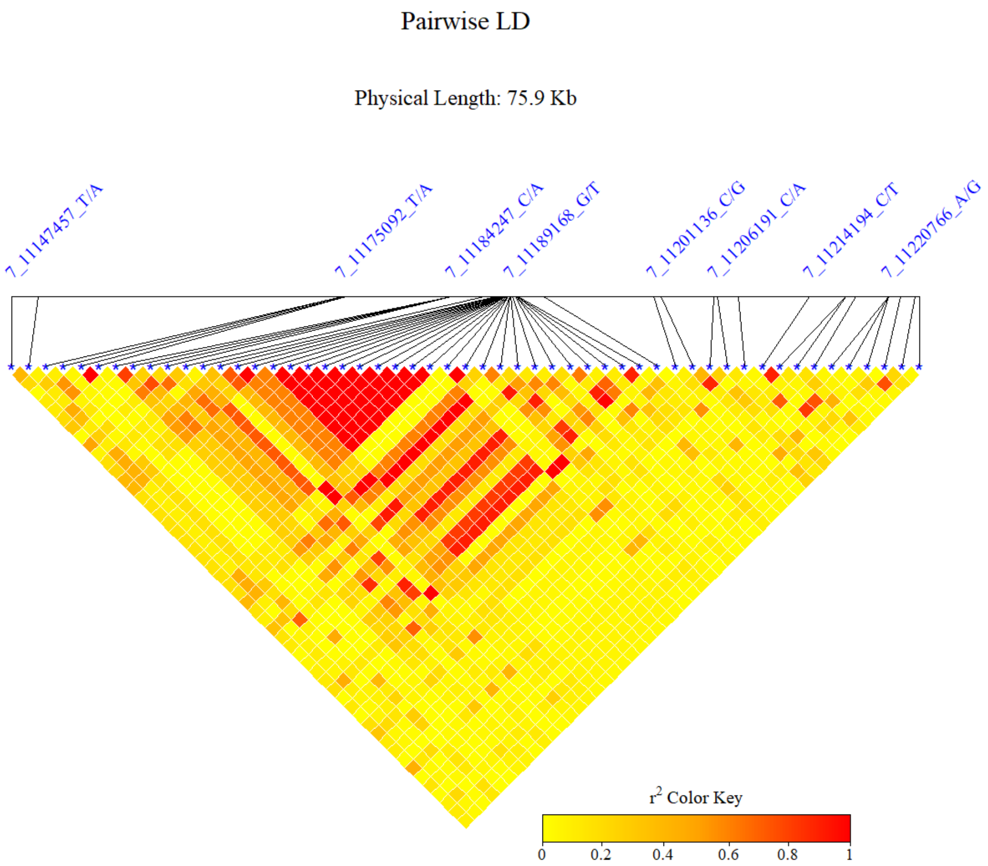

Figure S13: Pairwise LD plot representing LD between pairs of variants on GGC7 from candidate region of *Helicobacter*. The variants are given as chromosome, position and reference/alternate alleles separated by "_".

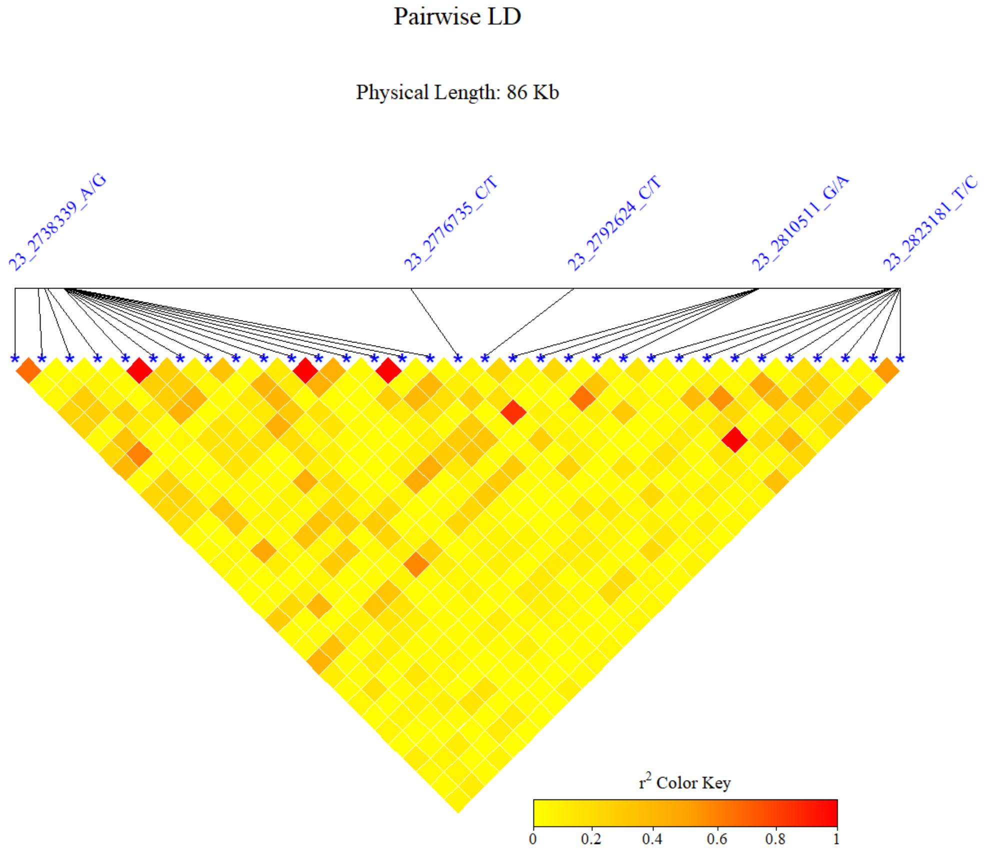

Figure S14: Pairwise LD plot representing LD between pairs of variants on GGC23 from candidate region of *Helicobacter*. The variants are given as chromosome, position and reference/alternate alleles separated by “_”.

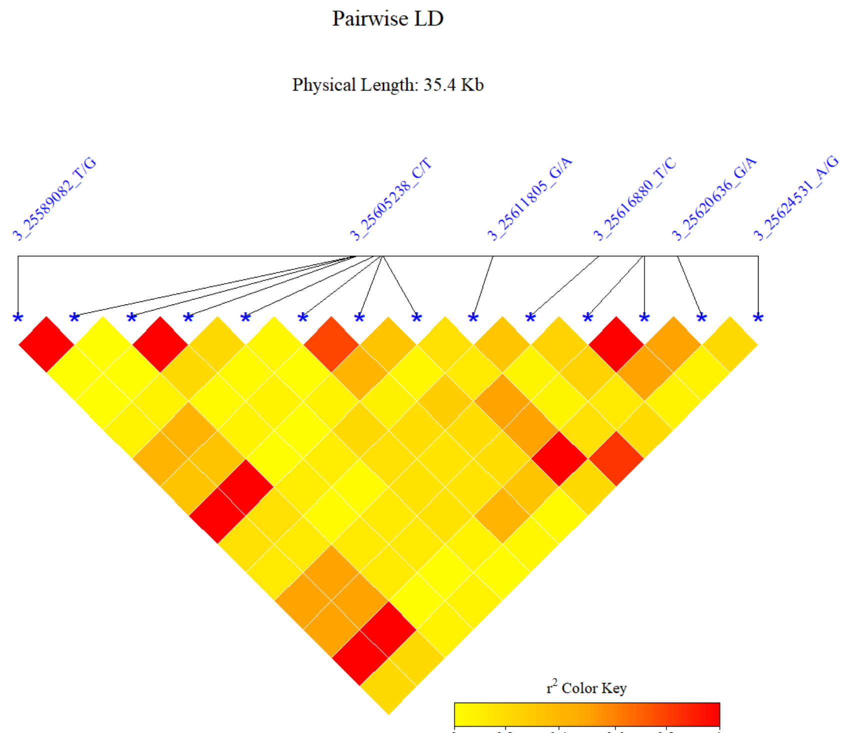

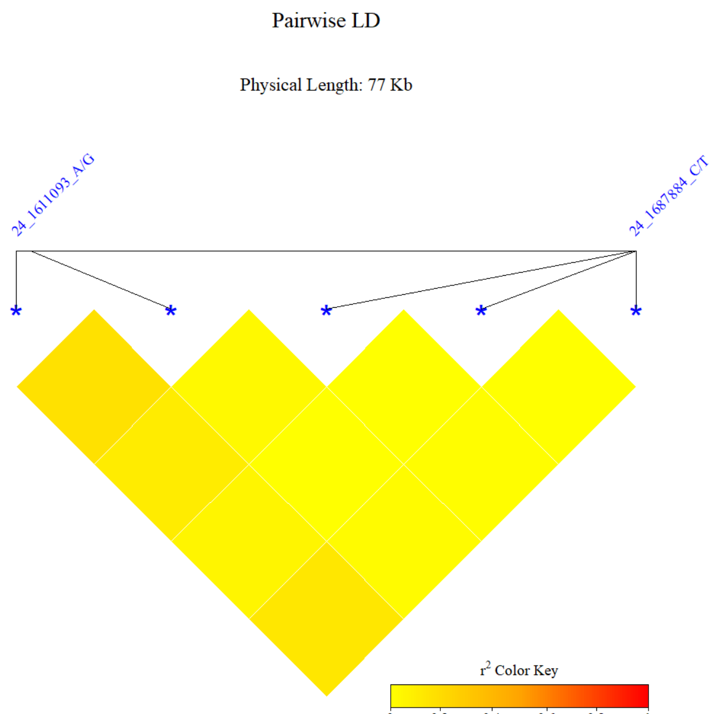

Figure S15: Pairwise LD plot representing LD between pairs of variants on GGC3 (left) and GGC24 (right) from candidate region of *Cloacibacillus*. The variants are given as chromosome, position and reference/alternate alleles separated by "_".

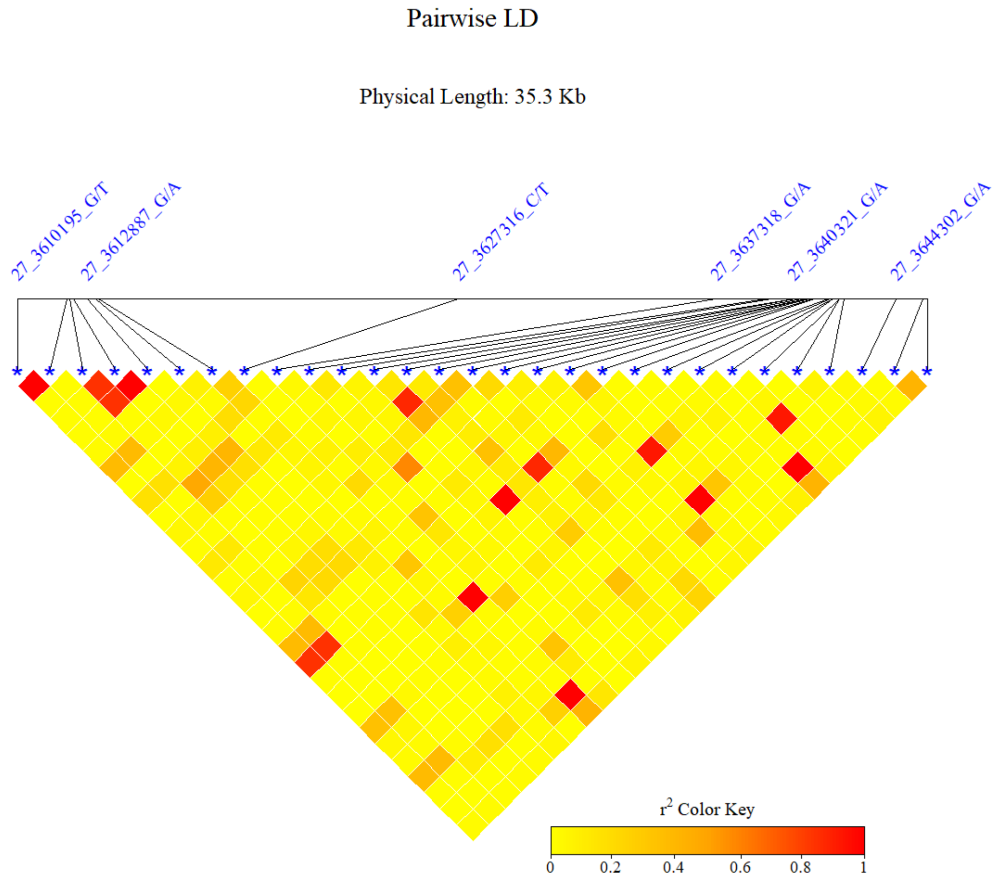

Figure S16: Pairwise LD plot representing LD between pairs of variants on GGC27 from candidate region of *Cloacibacillus*. The variants are given as chromosome, position and reference/alternate alleles separated by "_".

Table S1: List of genes identified in the candidate regions (50kb upstream/downstream of association) based on the significant and suggestive SNPs identified from GWAS. The genes mentioned here were considered for the KEGG pathway analysis.

Trait/genera	Ensembl Gene ID	Chromosome	Start position	End position	Gene symbol	Gene description	Gene biotype
Campylobacter	ENSGALG00000006461	2	8668167	8731922	*UBE3C*	ubiquitin protein ligase E3C	protein coding
Campylobacter	ENSGALG00000006477	2	8738366	8788229	*DNAJB6*	DnaJ heat shock protein family (Hsp40) member B6	protein coding
Campylobacter	ENSGALG00000051354	3	88927812	89141033			lncRNA
Campylobacter	ENSGALG00000051436	3	89110061	89117028			lncRNA
Campylobacter	ENSGALG00000010596	4	39167827	39202586	*TACR3*	tachykinin receptor 3	protein coding
Campylobacter	ENSGALG00000010601	4	39244685	39256258	*UFSP2*	UFM1 specific peptidase 2	protein coding
Campylobacter	ENSGALG00000045063	4	39256632	39264968	*LRP2BP*	LRP2 binding protein	protein coding
Campylobacter	ENSGALG00000010610	4	39265386	39318627	*SNX25*	sorting nexin 25	protein coding
Campylobacter	ENSGALG00000053147	4	72818132	72847666			lncRNA
Campylobacter	ENSGALG00000050988	4	72860057	72862975			lncRNA
Campylobacter	ENSGALG00000010879	8	26692555	26971644	*FGGY*	FGGY carbohydrate kinase domain containing	protein coding
Campylobacter	ENSGALG00000042168	8	26974401	26980880	*LOC100859636*	RING finger protein 170-like	protein coding
Campylobacter	ENSGALG00000010889	8	26991410	27014272	*HOOK1*	hook microtubule tethering protein 1	protein coding
Campylobacter	ENSGALG00000054726	8	27016860	27024977			lncRNA
Campylobacter	ENSGALG00000050045	8	27019347	27022914			protein coding
Campylobacter	ENSGALG00000051992	8	27026464	27032368	*CYP2J23*	cytochrome P450, family 2, subfamily J, polypeptide 23	protein coding
Campylobacter	ENSGALG00000001764	10	2482852	2579139	*HCN4*	hyperpolarization activated cyclic nucleotide gated potassium channel 4	pseudogene
Campylobacter	ENSGALG00000004004	11	7505176	7578698	*PHKB*	phosphorylase kinase regulatory subunit beta	protein coding
Campylobacter	ENSGALG00000006757	14	8412741	8686419	*XYLT1*	xylosyltransferase 1	protein coding
Campylobacter	ENSGALG00000051431	14	8714188	8720429	*RPS15A*	ribosomal protein S15a	protein coding
Campylobacter	ENSGALG00000044505	25	2602871	2608347	*SH2D2A*	SH2 domain containing 2A	protein coding
Campylobacter	ENSGALG00000040696	25	2606875	2615791	*PRCC*	papillary renal cell carcinoma (translocation-associated)	protein coding
Campylobacter	ENSGALG00000032847	25	2617131	2622609	*HDGF*	hepatoma-derived growth factor	protein coding
Campylobacter	ENSGALG00000030994	25	2623109	2624227	*MRPL24*	mitochondrial ribosomal protein L24	protein coding

Campylobacter	ENSGALG00000000576	25	2624337	2626380	*METTL25B*	ribosomal RNA adenine dimethylase domain containing 1	protein coding
Campylobacter	ENSGALG00000045721	25	2626869	2630717	*ISG20L2*	interferon stimulated exonuclease gene 20 like 2	protein coding
Campylobacter	ENSGALG00000029140	25	2633647	2636309	*CRABP2*	cellular retinoic acid binding protein 2	protein coding
Campylobacter	ENSGALG00000039072	25	2640456	2644375	*LOC425431*	dnaJ homolog subfamily A member 1-like	protein coding
Campylobacter	ENSGALG00000053624	25	2645368	2658858			lncRNA
Campylobacter	ENSGALG00000013239	25	2647908	2649620			pseudogene
Campylobacter	ENSGALG00000045469	25	2658942	2671332	*BCAN*	brevican	protein coding
Campylobacter	ENSGALG00000013234	25	2672117	2674826	*HAPLN2*	hyaluronan and proteoglycan link protein 2	protein coding
Campylobacter	ENSGALG00000013232	25	2675996	2680461	*RHBG*	Rh family B glycoprotein	protein coding
Campylobacter	ENSGALG00000054360	25	2696014	2698297			lncRNA
Cloacibacillus	ENSGALG00000030607	1	54386968	54542011	*CHST11*	carbohydrate sulfotransferase 11	protein coding
Cloacibacillus	ENSGALG00000046956	1	63649353	63660338			lncRNA
Cloacibacillus	ENSGALG00000025472	1	63754792	63754910	*5S_rRNA*	5S ribosomal RNA	rRNA
Cloacibacillus	ENSGALG00000047511	1	103588546	103589595			protein coding
Cloacibacillus	ENSGALG00000053698	1	103621663	103643070			lncRNA
Cloacibacillus	ENSGALG00000054509	1	131137495	131142444			lncRNA
Cloacibacillus	ENSGALG00000036877	1	154617566	154814374			lncRNA
Cloacibacillus	ENSGALG00000030691	3	25523064	25581617	*PPM1B*	protein phosphatase, Mg2+/Mn2+ dependent 1B	protein coding
Cloacibacillus	ENSGALG00000009973	3	25588912	25603301	*SLC3A1*	solute carrier family 3 member 1	protein coding
Cloacibacillus	ENSGALG00000009981	3	25603481	25620190	*PREPL*	prolyl endopeptidase-like	protein coding
Cloacibacillus	ENSGALG00000035800	3	25620462	25819300	*CAMKMT*	calmodulin-lysine N-methyltransferase	protein coding
Cloacibacillus	ENSGALG00000048468	3	25635900	25638628			lncRNA
Cloacibacillus	ENSGALG00000054461	4	52168201	52332950			lncRNA
Cloacibacillus	ENSGALG00000049729	4	52244222	52269370			lncRNA
Cloacibacillus	ENSGALG00000001331	24	1595777	1613311	*ST14*	suppression of tumorigenicity 14	protein coding
Cloacibacillus	ENSGALG00000001341	24	1652013	1665766	*ZBTB44*	zinc finger and BTB domain containing 44	protein coding
Cloacibacillus	ENSGALG00000001370	24	1687768	1698988	*ADAMTS8*	ADAM metallopeptidase with thrombospondin type 1 motif, 8	protein coding
Cloacibacillus	ENSGALG00000001055	27	3546258	3602488	*NSF*	N-ethylmaleimide sensitive factor, vesicle fusing ATPase	protein coding
Cloacibacillus	ENSGALG00000045889	27	3606663	3621331	*MEIOC*	meiosis specific with coiled-coil domain	protein coding

Cloacibacillus	ENSGALG00000001022	27	3623533	3631332	*CCDC43*	coiled-coil domain containing 43	protein coding
Cloacibacillus	ENSGALG00000035814	27	3631523	3641626	*DBF4B*	DBF4 zinc finger B	protein coding
Cloacibacillus	ENSGALG00000035673	27	3641617	3651847	*ADAM11*	ADAM metallopeptidase domain 11	protein coding
Cloacibacillus	ENSGALG00000053242	27	3648701	3648764	*gga-mir-12254*	gga-mir-12254	miRNA
Cloacibacillus	ENSGALG00000000997	27	3660178	3663464	*GJC3*	gap junction protein gamma 3	protein coding
Eisenbergiella	ENSGALG00000007278	14	9652802	9801024	*GRIN2A*	glutamate ionotropic receptor NMDA type subunit 2A	protein coding
Eisenbergiella	ENSGALG00000053538	14	9856027	9863285			lncRNA
Eisenbergiella	ENSGALG00000006363	15	8412346	8504251	*CABIN1*	calcineurin binding protein 1	protein coding
Eisenbergiella	ENSGALG00000006374	15	8525645	8536564	*TBX6*	T-box 6	protein coding
Eisenbergiella	ENSGALG00000052765	15	8539284	8547934			lncRNA
Eisenbergiella	ENSGALG00000006390	15	8549986	8561869	*CRKL*	CRK like proto-oncogene, adaptor protein	protein coding
Eisenbergiella	ENSGALG00000006422	15	8567577	8582523	*KLHL22*	kelch like family member 22	protein coding
Enterococcus	ENSGALG00000048821	2	72285407	72306386			lncRNA
Enterococcus	ENSGALG00000052411	2	72321292	72322521			protein coding
Enterococcus	ENSGALG00000051616	2	73909214	73909262			miRNA
Enterococcus	ENSGALG00000053224	2	73974581	73977953			lncRNA
Enterococcus	ENSGALG00000050810	12	19187212	19193743			lncRNA
Enterococcus	ENSGALG00000037841	12	19261785	19486230	*GRM7*	glutamate metabotropic receptor 7	protein coding
Enterococcus	ENSGALG00000029865	18	8590017	8744603			lncRNA
Enterococcus	ENSGALG00000049132	18	8697270	8707843			lncRNA
Enterococcus	ENSGALG00000048988	18	8714729	8719033			lncRNA
Enterococcus	ENSGALG00000040705	18	8739093	8784406			lncRNA
Enterotype	ENSGALG00000021567	21	6118469	6146506	*EPHA8*	EPH receptor A8	protein coding
Escherichia.Shigella	ENSGALG00000048736	8	12920099	12923043			lncRNA
Escherichia.Shigella	ENSGALG00000018336	8	12923059	12923154	*gga-mir-137*	gga-mir-137	miRNA
Escherichia.Shigella	ENSGALG00000034994	8	12961396	13078957			lncRNA
Escherichia.Shigella	ENSGALG00000005509	8	12966245	13290132	*DPYD*	dihydropyrimidine dehydrogenase	protein coding
Escherichia.Shigella	ENSGALG00000002319	23	4293444	4298380	*TEKT2*	tektin 2	protein coding
Escherichia.Shigella	ENSGALG00000053317	23	4299342	4306222			lncRNA
Escherichia.Shigella	ENSGALG00000043711	23	4307437	4329236	*AGO3*	argonaute 3, RISC catalytic component	protein coding
Escherichia.Shigella	ENSGALG00000002249	23	4331597	4354226	*AGO1*	argonaute 1, RISC catalytic component	protein coding
Escherichia.Shigella	ENSGALG00000039713	23	4358966	4370617	*AGO4*	argonaute 4, RISC catalytic component	protein coding
Escherichia.Shigella	ENSGALG00000002346	23	4371910	4385436	*CLSPN*	claspin	protein coding
Escherichia.Shigella	ENSGALG00000051443	23	4394410	4396671	*C1orf216*	chromosome 23 C1orf216 homolog	protein coding

Helicobacter	ENSGALG00000053852	1	72717945	72719564			lncRNA
Helicobacter	ENSGALG00000017295	1	72752038	72764874	*PTHLH*	parathyroid hormone like hormone	protein coding
Helicobacter	ENSGALG00000008152	7	11092899	11169151	*SPATS2L*	spermatogenesis associated serine rich 2 like	protein coding
Helicobacter	ENSGALG00000008155	7	11175038	11181067	*KCTD18*	potassium channel tetramerization domain containing 18	protein coding
Helicobacter	ENSGALG00000045898	7	11182555	11193986	*SGO2*	shugoshin 2	protein coding
Helicobacter	ENSGALG00000008185	7	11196756	11233011	*AOX1*	aldehyde oxidase 1	protein coding
Helicobacter	ENSGALG00000034756	23	2734269	2742975	*CTGFL*	connective tissue growth factor-like	protein coding
Helicobacter	ENSGALG00000001329	23	2743037	2816538	*EPB41*	erythrocyte membrane protein band 4.1	protein coding
Helicobacter	ENSGALG00000024318	23	2822852	2824408	*TMEM200B*	transmembrane protein 200B	protein coding
Parasutterella	ENSGALG00000015403	1	90418011	90635463	*EPHA3*	EPH receptor A3	protein coding
Parasutterella	ENSGALG00000053901	1	90666063	90702770			lncRNA
Parasutterella	ENSGALG00000035461	1	188868223	188880800	*CHORDC1*	cysteine and histidine rich domain containing 1	protein coding
Parasutterella	ENSGALG00000054821	1	188891315	188922631	*NAALAD2*	N-acetylated alpha-linked acidic dipeptidase 2	protein coding
Parasutterella	ENSGALG00000017234	1	188927712	188953081	*FOLH1*	folate hydrolase 1	protein coding
Parasutterella	ENSGALG00000015556	4	80530710	80589183	*AFAP1*	actin filament associated protein 1	protein coding
Parasutterella	ENSGALG00000043448	4	80649049	80786524	*ABLIM2*	actin binding LIM protein family member 2	protein coding
Sutterella	ENSGALG00000017136	1	180268523	180278083	*GJB6*	gap junction protein beta 6	protein coding
Sutterella	ENSGALG00000048789	1	180296030	180296344			protein coding
Sutterella	ENSGALG00000022720	1	180296063	180296740	*GJB2*	gap junction protein beta 2	protein coding
Sutterella	ENSGALG00000017137	1	180313130	180314665	*GJA3*	gap junction protein alpha 3	protein coding
Sutterella	ENSGALG00000017139	1	180355361	180392875	*ZMYM2*	zinc finger MYM-type containing 2	protein coding
Enterotype	ENSGALG00000011088	5	46646127	46650779	*GSKIP*	GSK3B interacting protein	protein coding
Enterotype	ENSGALG00000011093	5	46651390	46670540	*AK7*	adenylate kinase 7	protein coding
Enterotype	ENSGALG00000033099	5	46671242	46711072	*PAPOLB*	poly(A) polymerase beta	protein coding
Enterotype	ENSGALG00000051850	5	46730626	46730684			miRNA
Enterotype	ENSGALG00000011116	5	46806027	46838896	*VRK1*	vaccinia related kinase 1	protein coding
Enterotype	ENSGALG00000004741	21	5956010	6053651	*EPHB2*	EPH receptor B2	protein coding
Enterotype	ENSGALG00000004771	21	6073556	6075682	*C1QB*	complement C1q B chain	protein coding
Enterotype	ENSGALG00000029669	21	6076987	6078857	*C1QC*	complement C1q C chain	protein coding
Enterotype	ENSGALG00000021569	21	6079357	6081398	*C1QA*	complement C1q A chain	protein coding
